# Epigenetic derepression converts PPARγ into a druggable target in triple-negative and endocrine-resistant breast cancers

**DOI:** 10.1038/s41420-021-00635-5

**Published:** 2021-09-27

**Authors:** Ser Yue Loo, Nicholas L. Syn, Angele Pei-Fern Koh, Janet Cheng-Fei Teng, Amudha Deivasigamani, Tuan Zea Tan, Aye Aye Thike, Shireen Vali, Shweta Kapoor, Xiaoyuan Wang, Jiong Wei Wang, Puay Hoon Tan, George W. Yip, Gautam Sethi, Ruby Yun-Ju Huang, Kam Man Hui, Lingzhi Wang, Boon Cher Goh, Alan Prem Kumar

**Affiliations:** 1 Cancer Science Institute of Singapore and Department of Biochemistry, Yong Loo Lin School of Medicine, National University of Singapore, Singapore, Singapore; 2 Genome Institute of Singapore, A*STAR, Singapore, Singapore; 3 Department of Haematology-Oncology, National University Cancer Institute, Singapore, Singapore; 4 Yong Loo Lin School of Medicine, National University of Singapore, Singapore, Singapore; 5 Cancer Science Institute of Singapore, National University of Singapore, Singapore, Singapore; 6 Department of Anatomy, Yong Loo Lin School of Medicine, National University of Singapore, Singapore, Singapore; 7 Division of Cellular and Molecular Research, National Cancer Centre Singapore, Singapore, Singapore; 8 Department of Pathology, Singapore General Hospital, Singapore, Singapore; 9 Cellworks Research India Pvt. Ltd., Bengaluru, India; 10 Department of Surgery, Yong Loo Lin School of Medicine, National University of Singapore, Singapore, Singapore; 11 Cardiovascular Research Institute (CVRI), National University Heart Centre, Singapore (NUHCS), National University Health System, Singapore, Singapore; 12 Department of Physiology, Yong Loo Lin School of Medicine, National University of Singapore, Singapore, Singapore; 13 Department of Pharmacology, Yong Loo Lin School of Medicine, National University of Singapore, Singapore, Singapore; 14 School of Medicine, College of Medicine, National Taiwan University, Taipei, Taiwan; 15 NUS Centre for Cancer Research (N2CR), Yong Loo Lin School of Medicine, National University of Singapore, Singapore, Singapore; 16 National University Cancer Institute, National University Health System, Singapore, Singapore; 17 Department of Haematology-Oncology, National University Hospital, National University Health System, Singapore, Singapore

**Keywords:** Breast cancer, Translational research

## Abstract

Clinical trials repurposing peroxisome proliferator-activated receptor-gamma (PPARγ) agonists as anticancer agents have exhibited lackluster efficacy across a variety of tumor types. Here, we report that increased *PPARG* expression is associated with a better prognosis but is anticorrelated with histone deacetylase (HDAC) 1 and 2 expressions. We show that HDAC overexpression blunts anti-proliferative and anti-angiogenic responses to PPARγ agonists via transcriptional and post-translational mechanisms, however, these can be neutralized with clinically approved and experimental HDAC inhibitors. Supporting this notion, concomitant treatment with HDAC inhibitors was required to license the tumor-suppressive effects of PPARγ agonists in triple-negative and endocrine-refractory breast cancer cells, and combination therapy also restrained angiogenesis in a tube formation assay. This combination was also synergistic in estrogen receptor-alpha (ERα)–positive cells because HDAC blockade abrogated ERα interference with PPARγ-regulated transcription. Following a pharmacokinetics optimization study, the combination of rosiglitazone and a potent pan-HDAC inhibitor, LBH589, stalled disease progression in a mouse model of triple-negative breast cancer greater than either of the monotherapies, while exhibiting a favorable safety profile. Our findings account for historical observations of de-novo resistance to PPARγ agonist monotherapy and propound a therapeutically cogent intervention against two aggressive breast cancer subtypes.

## Introduction

Breast cancers—the leading cause of cancer-related deaths among women—encompass a diverse and heterogeneous group of disease entities [1]. Patients diagnosed with the biologically aggressive triple-negative (TNBC) or endocrine-refractory subtypes often confront a bleak prognosis, in part because they respond poorly to cytotoxic chemotherapy regimens, and novel targeted agents have largely failed to encroach into the current therapeutic armamentarium.

The nuclear hormone receptor, peroxisome proliferator-activated receptor-γ (PPARγ), is the molecular target of the thiazolidinedione (TZD) class of drugs. Examples of TZD drugs include the FDA-approved oral anti-glycemic medications, rosiglitazone, and pioglitazone, whose safety profiles are well-established [2]. Upon ligand activation, PPARγ translocates from the cytoplasm into the nucleus and heterodimerizes with retinoic X receptor (RXR), and then binds to specific DNA sequences known as peroxisome proliferator response elements (PPREs) of target genes to modulate gene expression.

Regulatory networks orchestrated by PPARγ govern pleiotropic cellular processes related to energy metabolism, angiogenesis, cell cycle, and proliferation [3–5]. Ligand activation of PPARγ has also been found to exert anti-tumor effects in diverse preclinical models including breast cancer by inducing apoptosis, differentiation, cell growth inhibition and cell cycle arrest [3–9]. Clinically, the notion that PPARγ could function as a tumor suppressor was reinforced by large epidemiological studies which observed that diabetic patients receiving thiazolidinediones had up to ~33% lowered risk for developing certain malignancies [10, 11]. These observations galvanized a few small clinical trials to examine the antitumor effects of PPARγ agonists in human malignancies. Unfortunately, TZDs appeared to only exert modest antiproliferative effects against pleomorphic/myxoid round-cell liposarcomas [9], but patients with heavily pretreated advanced carcinomas including breast cancer [12], colorectal cancer [13], and prostate cancer [14] appeared to exhibit primary resistance to PPARγ targeted therapy. Enthusiasm for the repurposing of anti-diabetic TZD drugs as anti-cancer therapeutics therefore waned.

In retrospect, tumors from heavily pretreated patients often harbor extensive epigenomic alterations, which are increasingly appreciated to constitute a major driving force for therapy resistance [15–20]. This is clinically supported by a growing body of literature that histone deacetylase (HDAC) inhibitors, such as vorinostat, may reinstate or enhance the sensitivity of cancer cells to systemic and radiation treatments [21]. It is therefore plausible that epigenetic changes could have accounted for the lack of response to TZD monotherapy in the few phase II breast cancer trial undertaken to date [12], which recruited patients with chemoresistant metastatic disease who had undergone multiple lines of prior treatments. In this study, we report that aberrant HDAC activities impart resistance to TZDs via canonical and non-canonical mechanisms. As a corollary, we demonstrated that relieving these repressive circuitries with clinically approved or experimental HDAC inhibitors is essential to convert PPARγ into a tractable therapeutic target and that combination therapy elicits synergistic antitumor effects against preclinical models of TNBC and endocrine-refractory breast cancer. These results evince PPARγ as a rational drug target in breast malignancies and should rekindle efforts to repurpose TZD drugs as anti-cancer therapies.

## Results

### PPARγ expression correlates with improved survival but is anticorrelated with HDAC1/2 expression

**Fig. 1:**
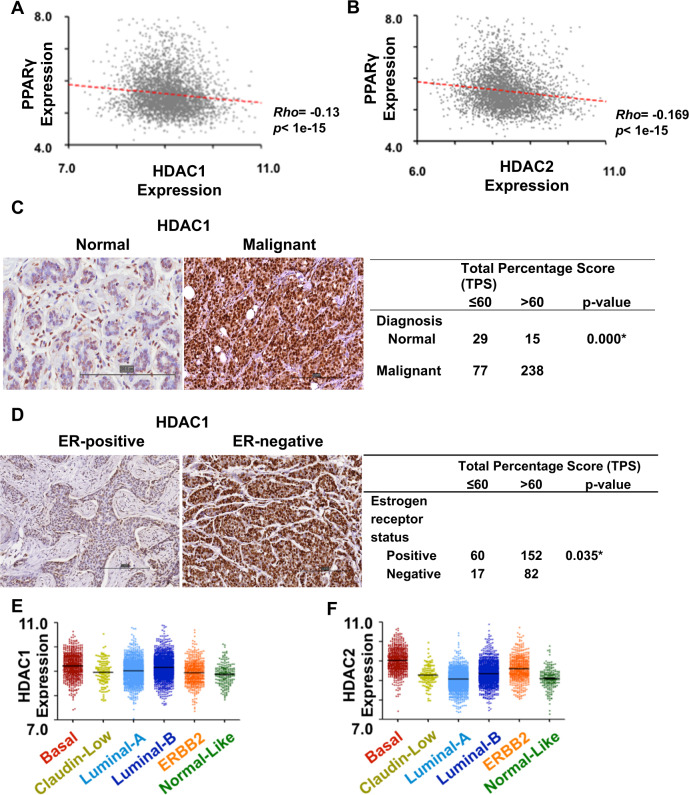
HDAC and PPARγ gene expression in breast cancer. **A** Correlation plot of PPARγ and HDAC1 expression. **B** Correlation plot of PPARγ and HDAC2 expression. **A**–**B** Spearman correlation test was performed, and the corresponding Rho and *p* values are shown next to the dot plots, *n* = 3992. **C** Left: HDAC1 immunostaining in normal and malignant tissues. A higher percentage of cells stained with HDAC1 was observed in malignant breast tissues compared to normal ductal tissues. Right: Statistics of HDAC1 expression between normal and malignant breast tissues. **D** Left: HDAC1 immunostaining in breast tissue showing nuclear localization. A higher percentage of cells stained with HDAC1 was observed in the IDC case having ER-negative status compared to the IDC case having ER-positive status. Right: Statistics of HDAC1 expression between IDC cases with ER-negative and positive statuses. **E** HDAC1 gene expression in breast cancer subtypes. **F** HDAC2 gene expression in breast cancer subtypes. **E**–**F** Color code: Basal, Maroon; Claudin-low, Yellow; Luminal-A, light blue; Luminal-B, dark blue; Normal-like, green.

Our first step was to deconvolute the clinicopathological and molecular correlates of PPARγ and HDAC expression in breast cancer. To this end, we leveraged 26 publicly available transcriptomic datasets comprising 3992 specimens and an in-house cohort of 390 tissue microarrays. Using random-effects meta-analysis on a subsample of 2151 primary breast cancer patients with overall survival data, we found high PPARγ mRNA expression (stratified by study-specific medians) to be associated with improved prognosis (inverse variance-weighted HR = 0.84; 95% CI: 0.71–0.99; *P* = 0.036) (Figs. S1A and S2A), which lends support to the notion that PPARγ acts as a tumor suppressor gene in breast cancer [3–9]. Furthermore, PPARγ was inversely correlated, albeit somewhat weakly, with the expression of HDAC1 and HDAC2 (Spearman rho = –0.13 [*P* < 1.0 × 10^−15^] and –0.17 [*P* < 1.0 × 10^−15^] respectively (Fig. 1A, B), suggesting that PPARγ could be directly or indirectly under the negative regulation of these class I histone deacetylases.

We also investigated whether any molecular subtypes or clinicopathological traits correlate with higher levels of HDAC expression, which could provide a set of enrichment or eligibility criteria for patient selection in clinical trials (Supplementary Results: Part S1). Patients with a higher percentage of tumor cells positively immune-stained for HDAC1 and HDAC2 expressions were more likely to experience shorter disease-free survival, as well as higher T and N stages, and triple-negative pathology (Fig. 1C, D, S3A, B). Next, we used single-sample gene set enrichment analysis (ssGSEA) [22] to assign intrinsic breast cancer subtypes to individual transcriptomes in our aggregated database, based on the similarities between their expression profiles with those of published molecular signatures [23]. Consistent with immunohistochemical analyses, basal-like breast cancers exhibited significantly higher HDAC1 and HDAC2 expression compared to other subtypes (*P* = 4.9 × 10^−19^ and *P* = 4.5 × 10^−156^, respectively), while luminal A and luminal B cases also featured higher HDAC1 expression (*P* = 6.2 × 10^−8^ and *P* = 8.7 × 10^−21^, respectively) compared with non-luminal cases (Fig. 1E, F).

### HDAC inhibition enhances PPARγ expression, acetylation, and PPRE activity

**Fig. 2:**
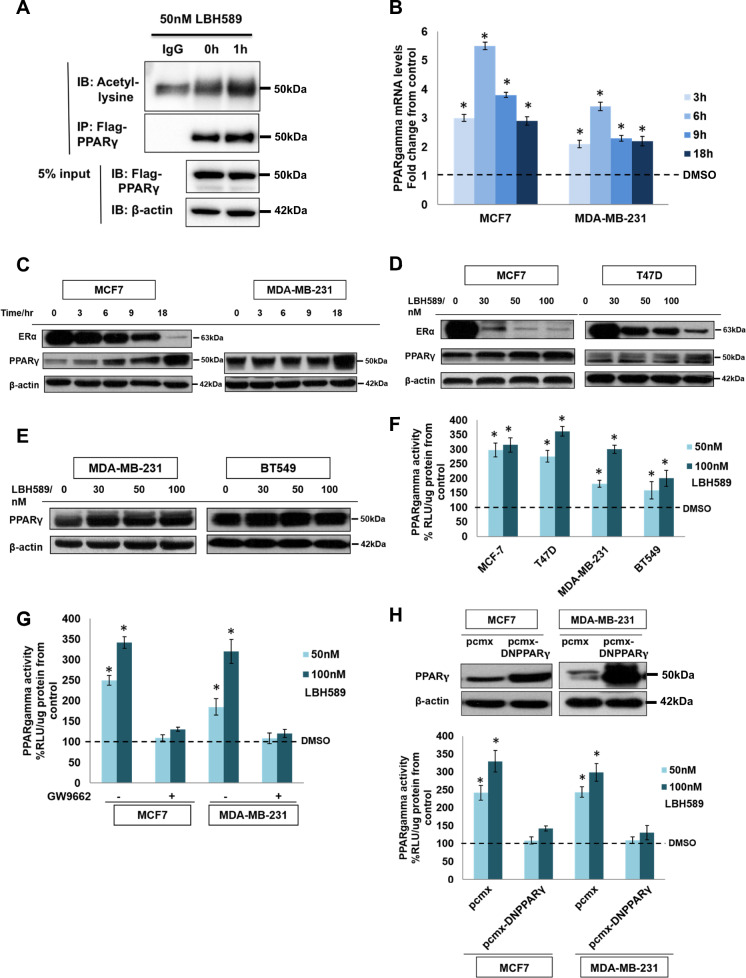
Increase in PPARγ acetylation status, activity, and protein expression by LBH589 treatment in breast cancer cell lines. **A** Top: Immunoprecipitation of PPARγ in MCF7 cells treated with 50 nM LBH589 for 1 h. PPARγ-FLAG was immunoprecipitated from cell lysates and immunoblot analysis was conducted for acetyl-lysine. Bottom: 5% of protein used for IP was run by Western blot to confirm unchanged levels of PPARγ following LBH589 treatment. **B** RT-PCR analysis of relative PPARγ mRNA levels normalized to 18 S mRNA levels. MCF7 and MDA-MB-231 cells were subjected to 50 nM LBH589 for 0, 3, 6, 9, and 18 h. Results are expressed as fold changes from control. Values represent mean ± SD, *n* = 3, *P < 0.05 vs. control. DMSO is added as vehicle control in 0 h condition. **C** Western blot analysis of MCF-7 and MDA-MB-231 cells treated for 0, 3, 6, 9, and 18 h with 50 nM LBH589. **D** Western blot analysis of MCF-7 and T47D cells treated with 0, 30, 50, and 100 nM of LBH589 for 24 h. **E** Western blot analysis of MDA-MB-231 and BT-549 cells treated with 0, 30, 50, and 100 nM of LBH589 for 24 h. (**F**) PPARγ activity of MCF-7, T47D, MDA-MB-231, and BT549 cells treated with 0, 50, and 100 nM LBH589 for 16 h. Values represent mean ± SD, *n* = 3, *P < 0.05 vs. control. DMSO is added as vehicle control in 0 nM LBH589 condition. **G** PPARγ activity of MCF-7 and MDA-MB-231 cells treated with 0, 50, and 100 nM LBH589 for 16 h+/− 4 h pre-incubation of 10 µM GW9662. Values represent mean ± SD, *n* = 3, *P < 0.05 vs. control. DMSO is added as vehicle control in 0 nM LBH589 condition. **H** Top: Western blot of MCF-7 and MDA-MB-231 cells transfected with empty vector pcmx or dominant-negative PPARγ mutant. Bottom: PPARγ activity of transfected cells treated with 0, 50, and 100 nM LBH589 for 16 h. Values represent mean ± SD, *n* = 3, *P < 0.05 vs. control. DMSO is added as vehicle control in 0 nM LBH589 condition.

If causal mechanisms are implicated in the negative correlation between HDAC1/2 and PPARG expression, relieving these repressive machinations with an HDAC inhibitor should have the effect of increasing PPARγ expression and activity. Within the first hour of incubating an estrogen receptor alpha-positive (ER+) breast cancer cell line, MCF7, in 50 nM of a potent pan-HDAC inhibitor, LBH589, we observed a marked initial accumulation of acetylated PPARγ, despite overall levels of FLAG-tagged PPARγ remaining unchanged (Fig. 2A). The histone deacetylases have been documented to catalyze the removal of acetyl moieties from nonhistone substrates to alter their function in malignant contexts [24, 25], as such, the rapid accumulation of acetylated PPARγ after LBH589 treatment likely reflects the post-translational deacetylation of PPARγ by class III (Sirtuin) HDAC activities [26, 27]. Subsequently, as evident from the 3 h mark, there was a steady and gradual rise in intracellular amounts of PPARγ mRNA and protein (Fig. 2B, C)—the temporally-protracted nature of which is in keeping with the better-known function of HDAC1/2 to modulate gene transcription through modifying chromatin accessibility. A dose-dependent effect, in which increasing concentrations of LBH589 were monotonically associated with the extent of PPARγ upregulation, was also observed in a variety of representative ER+ and triple-negative (TNBC) breast carcinoma cell lines (Fig. 2D, E). Finally, these transcriptional changes could also be affected using another clinically approved HDAC inhibitor, vorinostat (SAHA), and an experimental inhibitor, droxinostat (Fig. S4A, B), thus confirming a class effect. Taken together, the afore set of observations causally establish that PPARγ is under the negative regulation of HDACs in breast cancer.

Since we found that pan-HDAC blockade promotes the accumulation of acetylated PPARγ, which has been purported to possess heightened transcriptional activity [26, 27], our next investigations were to ascertain whether LBH589 treatment enhances PPARγ-mediated transcription. We took advantage of the fact that downstream genes targeted by this transcription factor contain peroxisome proliferator response elements (PPRE), where it binds. Hence, to probe PPARγ transcriptional activity, we employed a dual-luciferase reporter construct containing three PPREs from the rat acyl-CoA oxidase promoter that is under the control of the herpes simplex virus thymidine kinase promoter. Incubation with LBH589 dose-dependently enhanced the PPRE-luciferase reporter in MCF7, T47D, MDA-MB-231, and BT549 breast cell lines, indicating that HDAC blockade also augments PPARγ activity (Fig. 2F). To provide assurance that the enhanced bioluminescence is attributable to PPARγ receptor activation rather than some off-target consequence of pan-HDAC inhibition, we repeated the luciferase assays but modified the experiments to eliminate the contribution of PPARγ (Supplementary Results: Part S2).

### HDAC blockade unchains PPARγ-mediated anti-proliferative and anti-angiogenic signaling

**Fig. 3:**
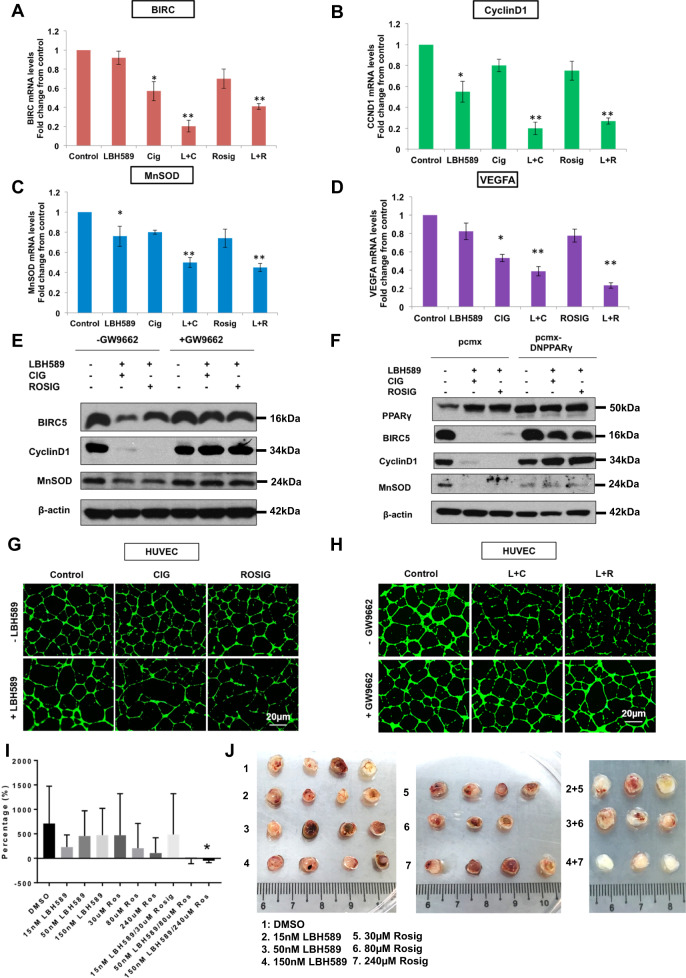
In vitro sensitization of breast cancer cells to PPARγ ligands and inhibition of angiogenesis in HUVECs by combination with LBH589. **A** MDA-MB-231 cells were subjected to 50 nM LBH589, 20 µM ciglitazone, 60 µM rosiglitazone, or a combination of LBH589+ ciglitazone /rosiglitazone for 24 h. RT-PCR analysis of relative *BIRC5* mRNA levels normalized to 18 S mRNA levels. **B** RT-PCR analysis of relative *Cyclin D1* mRNA levels normalized to 18 S mRNA levels. **C** RT-PCR analysis of relative *MnSOD* mRNA levels normalized to 18S mRNA levels. **D** RT-PCR analysis of relative *VEGFA* mRNA levels normalized to 18 S mRNA levels. Results are expressed as fold changes from control. Values represent mean ± SD, *n* = 3, *P < 0.05 vs. control, **P < 0.005 vs. control. **E** Western blot of MDA-MB-231 treated with 50 nM LBH589, 20 µM ciglitazone, 60 µM rosiglitazone, or a combination of both for 24 h+/− 4 h pre-incubation of 10 µM GW9662. DMSO is added as vehicle control in conditions of the absence of drugs. **F** Western blot of MDA-MB-231 transfected with pcmx or pcmx-DN PPARγ and treated with a combination of 50 nM LBH589 and 20 µM ciglitazone/60 µM rosiglitazone for 24 h. DMSO is added as vehicle control in conditions of the absence of drugs. **G** Fluorescence microscopy images of tube formation of HUVECs treated with 50 nM LBH589, 20 µM ciglitazone, 60 µM rosiglitazone, or a combination of LBH589 and ciglitazone/rosiglitazone for 12 h (×100 magnification). **H** Fluorescence microscopy images of tube formations of HUVECs treated with a combination of LBH589 and ciglitazone/rosiglitazone for 12 h+/− 4 h pre-incubation of 10 µM GW9662 (×100 magnification). **I** Percentage changes in CAM vascular volumes after 48 h of single and combination treatments with LBH589 and rosiglitazone. One-way ANOVA was performed. Error bars represent SEM. No significant changes in vascular volumes were obtained for single treatments of LBH589 or rosiglitazone compared to DMSO. 150 nM LBH589/240 µM Rosiglitazone reduced MDA-MB-231 CAM vascular volume by over 700%. *P < 0.05. **J** Images of MDA-MB-231 tumors formed on CAM and treated with DMSO, LBH589, or rosiglitazone for 48 h. No significant changes in tumor sizes were observed. The combined treatment of LBH589 and rosiglitazone produced significantly whiter tumors devoid of vascular membranes.

Because PPARγ has an extensive repertoire of downstream target genes while HDAC1/2 alters chromatin accessibility and gene expression on a genome-wide scale, pharmacological modulation of these pleiotropic regulators of gene transcription would invariably perturb a myriad of biological pathways simultaneously. To sieve out anticancer mechanisms efficiently, we began by conducting in silico simulations using a validated systems pharmacology model [28–38] (Supplementary Results: Part S3). We then validated these bioinformatics predictions through RT-qPCR and in vitro angiogenesis assays (Supplementary Results: Part S3**)**. Since the quintessential proangiogenic growth factor, VEGF-A was delineated through systems biology and RT-qPCR to be downregulated in a greater-than-additive manner by combination treatment, we extended our investigations to determine whether this translates to any functional anti-angiogenic effect. Combinatorial treatment using an HDAC inhibitor (LBH589) and a PPARγ agonist (rosiglitazone or ciglitazone) was found to significantly curb the number of well-defined capillary-like tubes and branching points formed by human umbilical vein endothelial cells (HUVEC) [39] in an endothelial tube formation assay, compared with either of the monotherapies (Fig. 3G and S5G–H). Conversely, when the assay was repeated by pre-treating the matrigel with a non-competitive PPARγ antagonist, the synergistic effects of the PPARγ agonist/HDAC inhibitor combination therapy against angiogenesis were abolished, thus confirming that the anti-vasculogenic effects are mediated by PPARγ activities (Fig. 3H and S5I–J). A chick chorioallantoic membrane (CAM) experiment was conducted to examine the effects of combination treatment on tumor vascularization in vivo. Treatment of the MDA-MB-231 CAM tumor with LBH589 did not have any significant effects on the tumor volumes compared to the DMSO control. However, a significant difference in vascular volume was observed from the combination treatment of 50 nM LBH589/80 µM rosiglitazone and 150 nM LBH589/240 µM rosiglitazone compared to individual drugs alone (Fig. 3I). The effects of the combination treatment could be observed in the MDA-MB-231 tumors excised from CAM, producing much whiter tumors devoid of vasculature compared to the DMSO control or single treatments (Fig. 3J).

### Synergistic effects of HDAC inhibitors and PPARγ agonists against TNBC cells

**Fig. 4:**
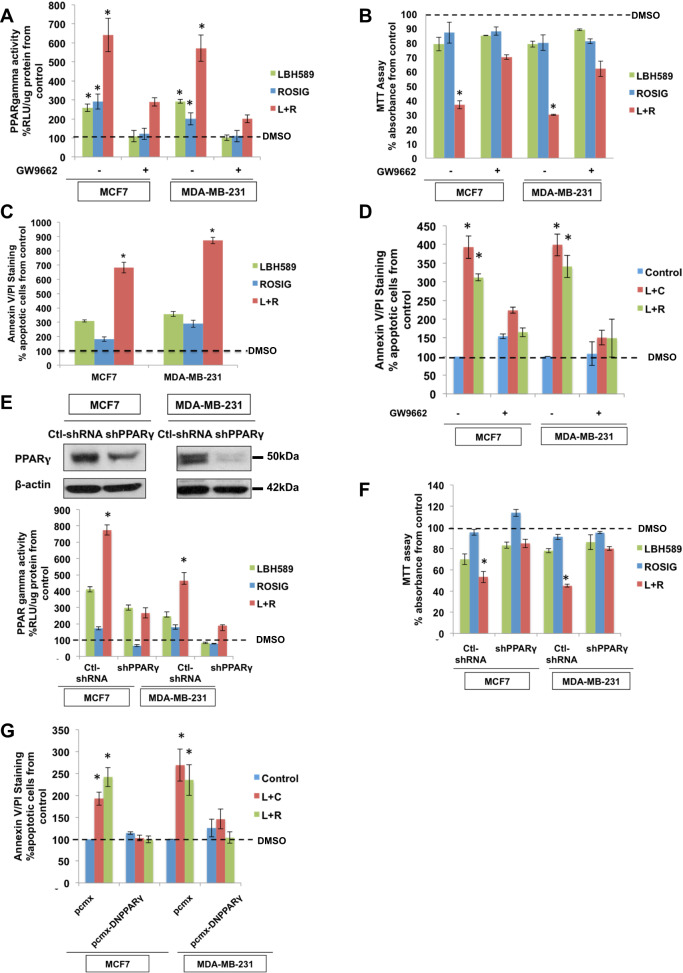
In vivo sensitization of breast cancer cells to PPARγ ligands upon the combination with LBH589. **A** PPARγ activity of MCF-7 and MDA-MB-231 cells treated with 50 nM LBH589, 60 µM rosiglitazone, or a combination of both for 16 h+/− 4 h pre-incubation of 10 µM GW9662. Values represent mean ± SD, *n* = 3, *P < 0.05 vs. control. DMSO is added as vehicle control in conditions of the absence of drugs. **B** Cell viability assay of MCF-7 and MDA-MB-231 cells treated with 50 nM LBH589, 60 µM rosiglitazone, or a combination of both for 24 h+/− 4 h pre-incubation of 10 µM GW9662. Values represent mean ± SD, *n* = 3, *P < 0.05 vs. control. DMSO is added as vehicle control in conditions of the absence of drugs. **C** Annexin V/PI staining assay of MCF-7 and MDA-MB-231 cells treated with 50 nM LBH589, 60 µM rosiglitazone, or a combination of both for 24 h. DMSO is added as vehicle control in conditions of the absence of drugs. **D** Annexin V/PI staining assay of MCF-7 and MDA-MB-231 cells treated with a combination of 50 nM LBH589 and 20 µM ciglitazone/60 µM rosiglitazone for 24 h+/− 4 h pre-incubation of 10 µM GW9662. Values represent mean ± SD, *n* = 3, *P < 0.05 vs. control. DMSO is added as vehicle control in conditions of the absence of drugs. **E** (Top): Western blot of MCF-7 and MDA-MB-231 cells transfected with control shRNA or shPPARγ. (Bottom): PPARγ activity of MCF-7 and MDA-MB-231 cells transfected with control shRNA or shPPARγ and treated with 50 nM LBH589, 60 µM rosiglitazone, or a combination of both for 16 h. Values represent mean ± SD, *n* = 3, *P < 0.05 vs. control. DMSO is added as vehicle control in conditions of the absence of drugs. **F** Cell viability assay of MCF-7 and MDA-MB-231 cells transfected with control shRNA or shPPARγ and treated with 50 nM LBH589, 60 µM rosiglitazone, or a combination of both for 24 h. Values represent mean ± SD, *n* = 3, *P < 0.05 vs. control. DMSO is added as vehicle control in conditions of the absence of drugs. **G** Annexin V/PI staining assay of MCF-7 and MDA-MB-231 cells transfected with control pcmx or DN PPARγ and treated with a combination of 50 nM LBH589 and 20 µM ciglitazone/60 µM rosiglitazone for 24 h. Values represent mean ± SD, *n* = 3, *P < 0.05 vs. control. DMSO is added as vehicle control in conditions of the absence of drugs.

Our upstream analyses had indicated that TNBC tumor specimens expressed higher levels of HDACs which, in turn, curtails PPARγ-mediated anticancer signaling pathways, suggesting that HDAC inhibition may be required to reverse the brakes on PPARγ-mediated anticancer effects. As such, our subsequent investigations homed in on the in vitro effects of combinatorial therapy involving HDAC inhibitors and PPARγ agonists against TNBC cells. A representative TNBC cell line, MDA-MB-231, was treated with various combinations of a PPARγ agonist (rosiglitazone or ciglitazone) and an HDAC inhibitor (LBH589, vorinostat, or droxinostat) for 24 h, and synergistic interactions were quantified based on the Chou-Talalay model [40–42]. Consistent with our preceding observations, these drug pairs exhibited synergistic combinatorial indices (Table S1), increasing PPARγ-dependent transcription of PPRE-luciferase reporter genes (Fig. 4A, S6A), lending further credence to the notion that PPARγ activity underlies the antiproliferative changes and induction of apoptosis. Cell viability was drastically impaired and the percentage of Annexin V/PI-stained cells undergoing apoptosis was enhanced more profoundly under combination therapies compared with individual single agents (Fig. 4B–D, S6B). In contrast, both pharmacological or genetic ablation of PPARγ activity abrogated the combinatorial synergy, establishing that the cytotoxic effects of combination therapy are attributable to ligand-dependent PPARγ activation rather than off-target effects of HDAC blockade (Supplementary Results: Part S4**)**.

### HDAC blockade restores sensitivity to thiazolidinediones in ER+ and endocrine-refractory breast cancer cells

Intriguingly, among the plurality of transcriptional changes invoked by HDAC inhibition, we observed that ERα receptor expression was also dose-dependently suppressed by LBH589 in the ER-positive cell lines MCF7 and T47D (Fig. 2C, D). This raised the question as to whether HDAC blockade could extinguish the antagonistic crosstalk between ERα and PPARγ, so as to license PPARγ-mediated cytotoxicity in ER-positive cells. Combining an HDAC inhibitor (LBH589, droxinostat, or vorinostat) with rosiglitazone or ciglitazone, we observed an increase in PPARγ activity significantly more pronounced than that achieved by the PPARγ agonist monotherapies (Fig. 4A and S7A, B), accompanied by prominent reductions in the fraction of viable cells and increases in Annexin V/PI-stained cells (Chou-Talalay combinatorial indices < 1.0; Fig. 4B, C and S7C, D). Pharmacological ablation or knockdown of PPARγ (using techniques as described in upstream analyses) abolished these synergistic anticancer effects, indicating that the combinatorial synergy of thiazolidinediones/HDAC inhibitors is contingent on PPARγ activity (Fig. 4B–D and 4F, G). These findings, therefore, demonstrate that—in contrast to a previous study which reported that MCF7 cells are resistant to rosiglitazone owing to ERα/PPARγ crosstalk [43]—the three ER-positive, hormone therapy-sensitive cell lines examined in our study are amenable to thiazolidinediones when the ERα interference with PPARγ-mediated effector cascades is abolished.

**Fig. 5:**
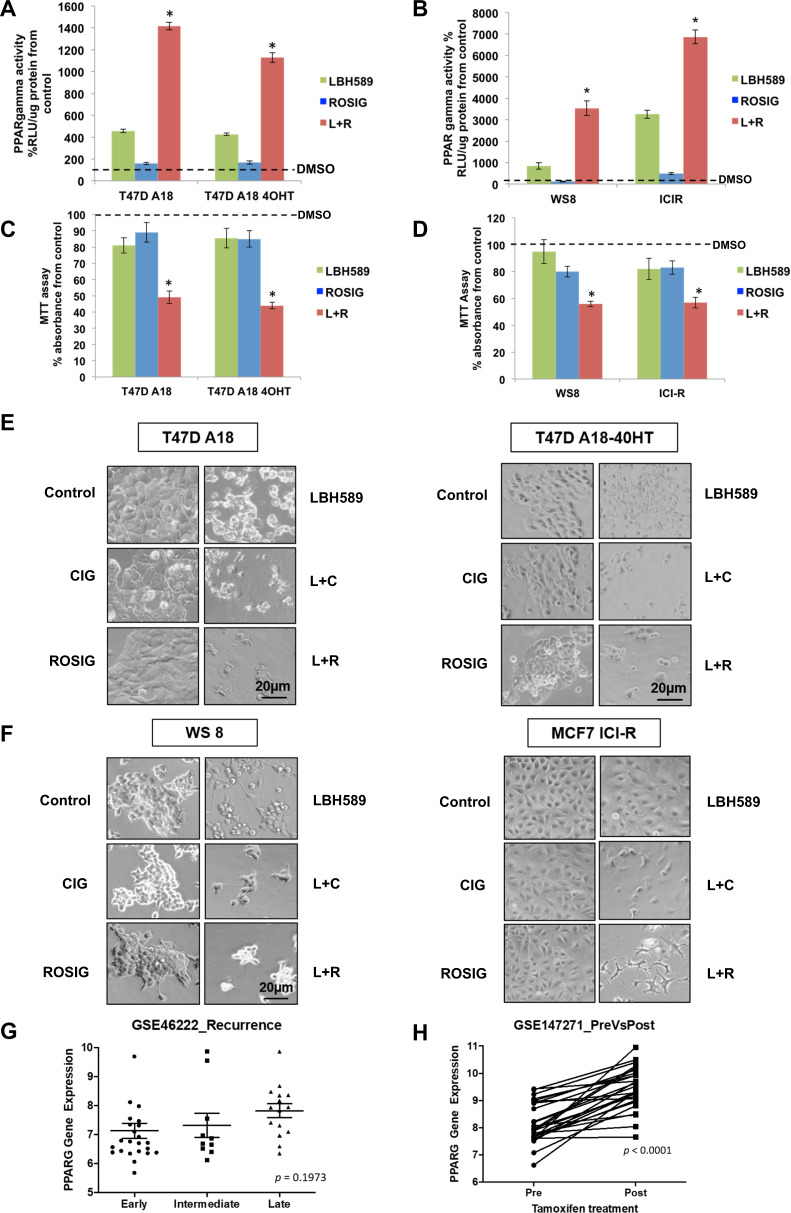
Combination of HDACi and PPARγ ligands increases sensitization of endocrine therapy-resistant cells. **A** PPARγ activity of T47D A18 and T47D A18 4OHT cells treated with 50 nM LBH589, 60 µM rosiglitazone, or a combination of both for 24 h. Values represent mean ± SD, *n* = 3, *P < 0.05 vs. control. DMSO is added as vehicle control in conditions of the absence of drugs. **B** PPARγ activity of WS8 and ICI-R cells treated with 50 nM LBH589, 60 µM rosiglitazone, or a combination of both for 24 h. Values represent mean ± SD, *n* = 3, *P < 0.05 vs. control. DMSO is added as vehicle control in conditions of the absence of drugs. **C** Cell viability assay of T47D A18 and T47D A18 4OHT cells treated with 50 nM LBH589, 60 µM rosiglitazone, or a combination of both for 24 h. Values represent mean ± SD, *n* = 3, *P < 0.05 vs. control. DMSO is added as vehicle control in conditions of the absence of drugs. **D** Cell viability assay of WS8 and ICI-R cells treated with 50 nM LBH589, 60 µM rosiglitazone, or a combination of both for 24 h. Values represent mean ± SD, *n* = 3, *P < 0.05 vs. control. DMSO is added as vehicle control in conditions of the absence of drugs. **E** Light microscopy images of T47D A18 (left) and T47D A18-4OHT (right) cells treated with 50 nM LBH589, 20 µM ciglitazone, 60 µM rosiglitazone, or a combination of LBH589 and ciglitazone/ rosiglitazone for 24 h (×200 magnification). **F** Light microscopy images of WS8 (left) and ICI-R (right) cells treated with 50 nM LBH589, 20 µM ciglitazone, 60 µM rosiglitazone, or a combination of LBH589 and ciglitazone/rosiglitazone for 24 h (×200 magnification). **G** PPARγ gene expressions from the GEO database were compiled for ER+ breast cancer patients with early, intermediate, and late recurrence. Anova test was conducted. **H** PPARγ gene expressions from the GEO database were compiled for ER + breast cancer patients pre-tamoxifen and post-tamoxifen treatment. Paired *t*-test was conducted.

Of greater clinical relevance, however, is the utility of this combinatorial strategy against endocrine treatment-refractory breast cancer, for which there remains a therapeutic void. To model clinical resistance to different estrogen-deprivation strategies, we exploited two additional ER-positive breast cancer sublines—MCF7:ICI-R and T47D:A18, which are resistant to fulvestrant and tamoxifen, respectively [44, 45]. Once again, PPARγ-mediated transcriptional activities were substantially enhanced upon combination treatment with LBH589 and rosiglitazone or ciglitazone compared to the monotherapies (Fig. 5A, B and S7E, F), and both cell lines also readily succumbed to combination therapy in a synergistic fashion compared to the individual drugs (Table S1, Fig. 5C–F and S7G, H). Next, we analyzed PPARγ expression level in ER+ breast cancer patients receiving tamoxifen monotherapy that have an early or late recurrence and found it to be higher in patients who had late recurrence (Fig. 5G). Furthermore, PPARγ gene expression was significantly increased in patients who have received tamoxifen therapy (Fig. 5H). These data, therefore, indicate that HDAC blockade could potentially transform PPARγ into a druggable target in endocrine therapy-refractory breast cancer, thus warranting further clinical appraisal of this combinatorial approach. At the same time, however, it must be acknowledged that high PPARγ expression alone does not guarantee robust anticancer responses to this combination strategy, as suggested by Fig. S8 where it can be seen that MCF10A and 12 A highly express PPARγ but are not greatly affected by combination treatment. This suggests that there may be additional mechanisms involved that warrant further elucidation in the future.

### Normal breast epithelial cells are spared from the synergistic effects of combination therapy

Because our previous experiments demonstrated that HDAC inhibition potentiates the cytotoxicity of thiazolidinediones against a range of breast cancer cell lines, we were curious as to whether similar synergistic effects are also experienced by non-malignant cells. To ensure comparability with our results involving breast carcinoma cells, we used their normal epithelial counterparts. In contrast to breast carcinoma cells, pan-HDAC blockade with LBH589 did not upregulate PPARγ expression or augment its activity in untransformed human breast epithelial cell lines MCF10A and MCF12A (Fig. S8A, B). This is likely explained by the notion that the deregulated HDAC axis which operates in breast carcinoma cells is not otherwise present in normal cells, hence attempting to relieve these repressive circuitries through HDAC blockade has no effect on PPARγ expression in non-cancerous cells. Furthermore, in contrast to our preceding experiments involving TNBC and ER-positive breast cancer cell lines, co-administration of LBH589 with rosiglitazone or ciglitazone did not synergistically impair the viability of non-cancerous cell lines compared with the individual monotherapies (Fig. S8C–F), suggesting that cancerous tissue—but not normal healthy tissue—are exquisitely vulnerable to the synergistic effects of combination therapy.

### Pharmacokinetic optimization and in vivo antitumor effects of rosiglitazone and LBH589

**Fig. 6:**
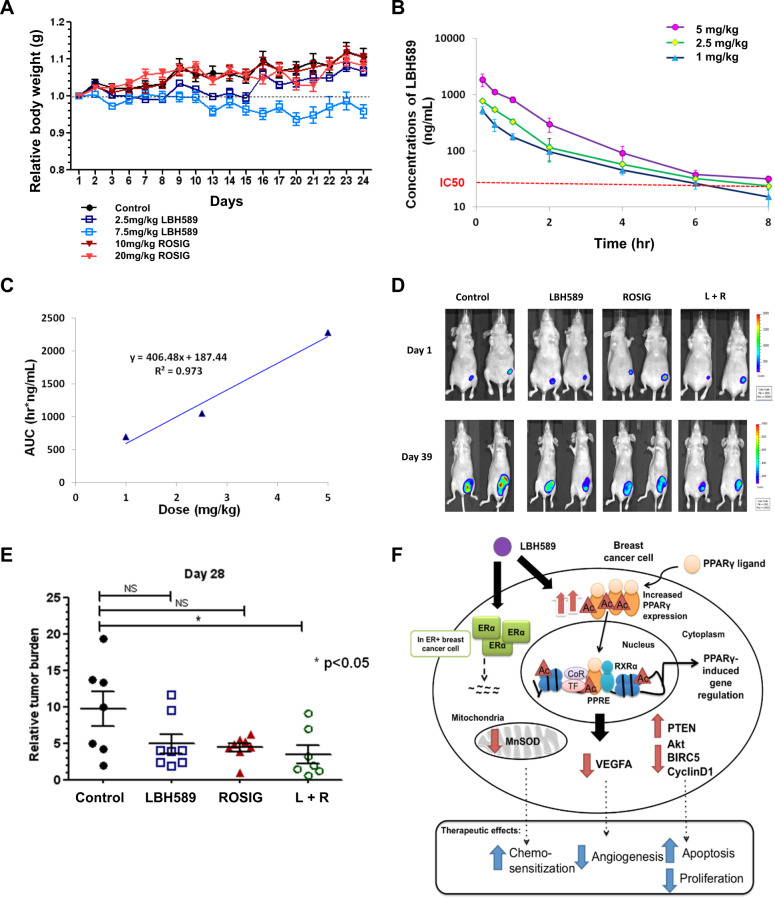
Pharmacokinetic optimization and in vivo antitumor effects of rosiglitazone and LBH589. **A** The body weight changes of mice for the toxicity study from day 1 until day 24. Mice were weighed five times a week prior to treatment. Mice were treated with LBH589 at 2.5 mg/kg or 7.5 mg/kg, Rosiglitazone at 10 mg/kg or 20 mg/kg. Control mice received 10% DMSO as a vehicle (*n* ≥ 7 animals per group). **B** Mice were dosed with 1 mg/kg, 2.5 mg/kg, and 5 mg/kg of LBH589, and serum concentrations of the drug were assessed at 10 min, 30 min, 1 h, 2 h, 4 h, 6 h, and 8 h post-treatment. Values represent mean ± SD (*n* = 3). **C** The association between doses of LBH589 and the values of AUC after intravenous injection of LBH589 at doses of 1 mg/kg, 2.5 mg/kg, and 5 mg/kg. **D** In vivo bioluminescence of tumors from the luciferase-expressing MDA-MB-231 cells in the abdominal mammary fat pad of mice at day 1 and 28, respectively. Luciferin substrate was injected into the intraperitoneal cavity of the mice and bioluminescence was examined after 10 min to obtain photon counts. Data for representative mice (two from each group) are shown. **E** Therapeutic efficacy of the LBH589 and Rosiglitazone or combination of both against mammary fat pad tumor growth in an orthotopic murine model at day 28. Mice were treated with 2.5 mg/kg LBH589, 10 mg/kg Rosiglitazone, or a combination of both from day 1 to day 28 when the tumors reached 10^7^ signals. Control mice received 10% DMSO as a vehicle solution. Relative tumor burden was calculated against the tumor bioluminescent signal just before treatment started (*n* ≥ 7 animals per group; mean ± SEM; *p ≤ 0.05 compared with negative control unless otherwise stated). **F** Model of combinational therapy of LBH589 and PPARγ ligands in breast cancer cells.

To define the tolerable dose range for downstream analyses, we monitored the bodyweight, activity levels, stooling habits, and other signs of distress in six-week-old female NCr nude mice (*n* = 39) randomized to receive LBH589 (2.5 mg/kg or 7.5 mg/kg) or rosiglitazone (10 mg/kg or 20 mg/kg) or control (10% DMSO vehicle) for five days per week of a three-week cycle. All treatment regimens were found to be well-tolerated, as none of the mice shed >10% of original bodyweight, and mice in all arms less those in the high-dose LBH589 group in fact gained bodyweight (Fig. 6A). Mice treated with 7.5 mg/kg LBH589 experienced less weight gain and softer stools compared to their counterparts treated with the lower dosage or placebo. Pharmacokinetic optimization studies revealed that the 2.5 and 5 mg/kg dosages sufficed to maintain serum exposure above the cellular IC50 of 50 nM for at least 6 h (Supplementary Results: Part S5).

Finally, we generated xenograft-bearing mice (*n* = 30) by orthotopic inoculation of luciferase-labeled, triple-negative MDA-MB-231 cells into their mammary fat pads. Mice were randomized to vehicle control (10% DMSO), LBH589 2.5 mg/kg, rosiglitazone 10 mg/kg, or a combination of both for five days per week for a total of four weeks. Treatment commenced when the disease burden in these animals reached an emission of 107 photons/s. Imaging assessments at day 28 showed that the relative tumor burden, which was calculated as the fold change in bioluminescent signal normalized to pre-treatment radiance levels, was 9.9 ± 6.2, 5.8 ± 3.7, 5.3 ± 0.8, and 3.0 ± 3.3 fold, respectively among mice in the control, LBH589, rosiglitazone, and combination treatment arms, respectively. Single-agent LBH589 or rosiglitazone was found to significantly curb tumor growth by –44.9% (*P* = 0.0322) and –51.7% (*P* = 0.0209), respectively compared to the control group. Remarkably, the combination regimen drastically stalled tumorigenesis by –69.3% compared to control (*P* < 0.0001), and to a greater extent than either the rosiglitazone (–36.4%; *P* = 0.0307) or LBH589 (–44.3%; *P* < 0.0001) monotherapies (Fig. 6D, E). Collectively, these results, therefore, establish a pharmacologically cogent combination regimen with an acceptable safety margin and justify further clinical studies in patients with triple-negative breast cancer.

## Discussion

Peroxisome proliferator-activated receptor-gamma (PPARγ) agonists—typified by the thiazolidinedione (TZD) class of oral anti-T2DM medications—have not lived up to original high expectations of being among the first non-oncological drugs to be repurposed as molecularly-targeted anticancer therapies. The few human efficacy trials conducted to date, in which TZDs were deployed as monotherapies in heavily-pretreated cancer patients, failed to demonstrate any objective clinical responses, causing many investigators to abandon this approach and casting uncertainty over the validity of PPARγ as a rational drug target [12–14]. However, like other molecular targeted therapies, PPARγ agonists may have limited effectiveness as single agents, especially in the setting of recurrent tumors, which are known to co-opt epigenetic mechanisms during the course of their evolution and adaption to anticancer regimens [15–20]. Our experiments herein show that overexpression of histone deacetylases (HDACs) in breast cancer cell lines and clinical tumor specimens blunts PPARγ-mediated effector cascades and identify a rational pairing of HDAC inhibitors and thiazolidinediones which yields synergistic tumor-suppressive and anti-angiogenic effects against a range of triple-negative and ER-positive breast cancer models.

In our experiments, the pairing of an HDAC inhibitor plus a PPARγ agonist synergistically induced apoptosis and curbed proliferation in a spectrum of triple-negative (TNBC) and ER-positive breast cancer cells, based on the Chou-Talalay statistical model for quantifying combinatorial interactions [40–42]. Intriguingly, the possible utility of this drug pair against ER-positive breast cancer was a serendipitous discovery motivated by the observation that ERα receptor expression was dose-dependently suppressed by LBH589 in ER-positive cell lines and HDAC blockade could therefore obviate its negative interference with PPARγ effector cascades. Crucially, the finding that combination therapy exerts potent cytotoxic effects against fulvestrant-refractory and tamoxifen-refractory subsets of ER-positive breast cancer cell lines merit further investigation because approximately 30–50% of women confront de-novo resistance to hormone therapy while another one-third of women who initially respond to therapy relapse with the endocrine-refractory disease within 15 years [46–48]. HDAC blockade was also found to augment TZD lethality against TNBC cells, and sensitized orthotopic xenograft models of TNBC to rosiglitazone, consistent with in vitro data. A reframing of our understanding of hormone resistance as the effect, rather than the cause, in breast cancer therapy resistance, could help us to better repurpose drugs.

Mechanistic and biochemical analyses consolidated several distinct mechanisms by which HDAC blockade unleashes PPARγ-mediated anticancer signaling cascades. We showed that HDAC inhibition (i) alleviated the transcriptional repression of PPARG, resulting in the upregulation of PPARγ mRNA and protein; (ii) prevents the post-translational modification of PPARγ by lysine deacetylation, resulting in a rapid initial accumulation of acetylated PPARγ, which is known to possess heightened transcriptional activity [26, 27]; (iii) enhanced the transactivation of PPRE target genes, due to the intrinsically higher activity of the acetylated form of PPARγ, as well as increased permissiveness of chromatin loci to PPARγ binding; and (iv) in the context of ER-positive cells, was found to interdict previously-described antagonistic crosstalk between nuclear receptors and resistance mechanism to PPARγ agonists [43] by culling ERα expression. Using a pipeline of computational prediction and experimental validation techniques, we discovered that the aforementioned set of transcriptional and post-translational mechanisms culminate in the perturbation of a small constituency of molecular pathways which likely mediate the synergistic anticancer effects of combination therapy. These transcriptional changes include a greater-than-additive downregulation of cell cycle genes encoding survivin (BIRC5) and cyclin D1 (CCND1), the pro-angiogenic growth factor VEGFA, and the redox enzyme manganese superoxide dismutase (SOD2), and upregulation of the PTEN tumor suppressor. In conjunction with the downregulation of VEGFA mRNA, we also proffered functional evidence that combination therapy restrained angiogenesis to a considerably greater extent than the individual monotherapies in capillary tube formation assays and CAM experiments. A summary of the functional effects induced by the combination treatment of LBH589 and PPARγ activation is illustrated in Fig. 6F.

To ensure the veracity of our conclusions, we replicated all experiments involving the combination therapy by modifying them in such a way as to ablate PPARγ activity using pharmacological (by pre-medicating cells with the non-competitive PPARγ antagonist, GW9662) and/or genetic (by silencing PPARγ expression using shRNA or transfection with a mutant dominant-negative receptor) techniques. Since the mode of action of PPARγ agonists and HDAC inhibitors are multifaceted and impinge on diverse signaling pathways, the use of pharmacological and genetic methods of perturbing PPARγ function enabled us to decouple the contribution of PPARγ and verify that the aforementioned transcriptional, anti-angiogenic, and cytotoxic effects observed during combination treatment were indeed engendered (at least in part) by PPARγ activity rather than some off-target effects from pan-HDAC blockade. Another notable aspect of our experimental design was the multiplicity of subtype-specific cancer and non-cancer cell lines as model systems, as well as the testing of various, approved and experimental PPARγ agonists and HDAC inhibitors to establish a pharmacological class effect, in order to improve the robustness and reproducibility of our findings.

Several lines of data indirectly suggest the tolerability of this combination therapy in human trials. Firstly, thiazolidinediones such as rosiglitazone remain one of the most widely prescribed class of anti-T2DM drugs with over two decades of pharmacovigilance data substantiating their safety profile, while the HDAC inhibitors vorinostat (SAHA) and LBH589 (panobinostat) have received FDA approval for the third-line treatment of primary cutaneous T cell lymphoma and multiple myeloma, respectively [15, 49–51]. Secondly, HDAC blockade was found to selectively upregulate PPARγ expression in malignant breast cells—but not their untransformed epithelial counterparts—rendering them profoundly susceptible to the combination regimen, while sparing non-cancerous tissue from the synergistic cytotoxicity of combination therapy.

In conclusion, these findings validate PPARγ as a targetable conduit in triple-negative and ER-positive breast cancers and open new opportunities for co-targeting PPARγ and HDACs using repurposed anti-diabetic (rosiglitazone) and anti-myeloma/lymphoma (LBH589 and SAHA) drugs. Serum concentrations expected to deliver meaningful pharmacological activity should be clinically attainable.

## Materials and methods

### Reagents

Roswell Park Memorial Institute (RPMI) 1640 medium, Dulbecco’s Modified Eagle Medium (DMEM), phosphate-buffered saline (PBS), fetal bovine serum (FBS), charcoal-stripped FBS, L-glutamine, and trypsin were purchased from Hyclone, UT, USA. Pepstatin A, phenylmethanesulfonyl fluoride (PMSF), leupeptin, propidium iodide (PI), crystal violet, bovine serum albumin (BSA), and mouse anti-β-actin monoclonal antibody was supplied by Sigma-Aldrich, LO, USA. Aprotinin was purchased from Applichem, Darmstadt, Germany. Rabbit anti-HuMnSOD and anti-HuER-alpha monoclonal antibodies were purchased from Upstate, NY, USA. Mouse anti-HuPPARγ, anti-HuCyclinD1, anti-HuSurvivin, and anti-Huβ-actin monoclonal antibodies were purchased from Santa Cruz Biotechnology, CA, USA. Stabilized goat anti-mouse horseradish peroxidase (HRP) and goat anti-rabbit horseradish peroxidase (HRP) were obtained from PIERCE, USA. Methanol and sodium dodecyl sulfate (SDS) were purchased from Merck, Darmstadt, Germany. Cell lysis buffer (1X) was from BD Pharmigen, USA.

### Cell lines and culture conditions

Breast cell lines MDA-MB-231, BT-549, MCF-7, T47D, MCF-10A, and MCF-12A (American Type Culture Collection, MD, USA) were used. Breast tumor cells lines were routinely maintained in RPMI 1640 for tumor cell lines supplemented with 10% FBS, 2mM L-glutamine, and gentamicin. MEGM (Mammary Epithelial Growth Medium) was used for normal epithelial cell lines supplemented with 10% FBS, 2mM L-glutamine, and a growth factor kit containing BPE, hydrocortisone, hEGF, insulin, and gentamicin (Lonza, MD, USA) in a 37 °C incubator with 5% CO_2_. Breast cancer cell lines T47D A18 and T47D A18 4OHT, WS8, and MCF7 ICI resistant cell lines were obtained from Jorden V. Craig [45]. Cells lines were routinely maintained in RPMI 1640 supplemented with 10% FBS, 2mM L-glutamine, and 0.05 mg/ml gentamicin in a 37 °C incubator with 5% CO_2_. Human umbilical vein endothelial cells (HUVECs) were obtained from Lobie P.E. (CSI, NUS, Singapore). Cells lines were routinely maintained in EBM (Endothelial Basal Medium) supplemented with 10% FBS, 2mM L-glutamine, and a growth factor kit containing hEGF, hydrocortisone, ascorbic acid, VEGF, hFGF-B, IGF-1, and gentamicin (Lonza, MD, USA) in a 37 °C incubator with 5% CO_2_.

### Treatment of Cells with LBH589, PPARγ ligands, and antagonist

For use in experiments, cells were trypsinized and seeded in 6-well plates (Falcon, NJ, USA) at 1.4 × 10^5^ cells per well and grown overnight on RPMI medium with 10% FBS. LBH589 was gifted by Boon-Cher Goh (CSI, NUS. Singapore) at stock solutions of 100 µM. Various concentrations of LBH589 were then prepared by diluting the stock solution with a complete medium to attain the desired final concentrations of 30 nM, 50 nM, and 100 nM. Stock solutions of 20 mM ciglitazone and 80 mM rosiglitazone (BIOMOL, USA) were prepared in DMSO. 20 µM ciglitazone and 60 µM rosiglitazone were prepared by diluting the stock solution with a complete medium to attain the desired final concentrations. For GW9662, a stock solution of 20 mM GW9662 (Cayman, USA) was prepared in DMSO, and a final concentration of 10 µM was used.

### Protein concentration determination and western blot analysis

Whole-cell lysates were prepared with RIPA lysis buffer containing 10 mM Tris-HCL pH7.4, 30 mM NaCl, 1 mM EDTA, 1% Nonidet P-40, supplemented with 1 mM sodium orthovanadate (Na3VO4), 1 µg/ml leupeptin, 1 µg/ml pepstatin A, 1 µg/ml aprotinin, and 1 mM PMSF before use. Protein concentration was determined for each sample, and equal amounts of protein were warmed at 37 °C in a water bath for 5 min with 1xSDS sample buffer and resolved by 12% SDS-PAGE. Thereafter, proteins were transferred onto nitrocellulose membranes, blocked for 1 h at room temperature with 5% non-fat milk, and incubated overnight at 4oC with the primary antibody. After probing with a secondary antibody for 1 h at 25 °C, protein bands were detected by using the Supersignal West Pico Chemiluminescent substrate kit. β-actin antibody was used as a loading control.

### RNA isolation, reverse transcription (RT), and real-time polymerase chain reaction (RT-PCR)

Total RNA was isolated from cells using TRIZOL reagent (Invitrogen, Carlsbad, CA, USA), as described in the manufacturer’s instructions with a DNAse treatment step incorporated into the protocol. Each RT reaction contained 2.5 µg of total RNA, 1× RT buffer, 5 mM MgCl_2_, 425 µM each of dNTPs, 2 µM random hexamers, 0.35U/µl RNase inhibitor, and 1.1U/µl MultiScribe™ reverse transcriptase and made up to 10 µl with sterile water. The RT reaction was carried out at 37 °C for 1 h. Five microlitres of the 10 µl cDNA reaction volume were used in real-time quantitative PCR using ABI PRISM 7500 (Applied Biosystems, Foster City, CA, USA). Normalization was endogenous 18S. Fluorescence was measured with the Sequence Detection Systems 2.0 software. PCR was performed in multiplex (both target and endogenous control co-amplified in the same reaction) with distinct fluorescent dyes. Primers and probes for human 18S, human PPARγ, BIRC5, CyclinD1, VEGFA, and MnSOD were purchased from Applied Biosystems (Assays on Demand).

### DNA and siMnSOD transfection

Plasmid pCMX-mPPARγ, a cDNA clone encoding the mouse PPARγ, was a generous gift from Dr. Ronald M. Evans, The Salk Institute for Biological Studies, San Diego, CA, USA. The PPARγ mutant (PPARγC126A/E127A) (PPARγ-DN) containing amino acid substitutions in the DNA binding domain that abolish binding to PPARγ response elements was kindly provided by Dr. Christopher K. Glass, UCSD, San Diego, CA, USA. Plasmid pcDNA flag PPARγ (plasmid 8895) was purchased from Addgene. The pcDNA3-MnSOD plasmid was kindly provided by Dr. Larry W. Oberley (University of Iowa, Iowa City, IA). Short hairpin RNA (shRNA) constructs against PPARγ were purchased from OriGene (OriGene Technologies, MD, USA).

shRNA1: CCTTCACTACTGTTGACTTCTCCAGCATT.

shRNA2: CAGTGGTTGCAGATTACAAGTATGACCTG.

shRNA3: TGAGAAGACTCAGCTCTACAATAAGCCTC.

shRNA4: TGACTTGAACGACCAAGTAACTCTCCTCA.

Negative control shRNA1: GCACTACCAGAGCTAACTCAGATAGTACT.

Negative control shRNA2: GCACTACCAGAGCTAACTCAGATAGTACT.

In vitro transfections were performed using LipofectAMINE 2000 (Invitrogen, Carlsbad, CA) following the manufacturer’s protocols.

### Luciferase assay

The luciferase reporter construct used was pPPRE-tk-Luc, which contains three PPREs from the rat acyl-CoA oxidase promoter under the control of the Herpes simplex virus thymidine kinase promoter (kind gift from Dr. Ronald M. Evans, The Salk Institute for Biological Studies, San Diego, CA, USA). 3× PPRE promoter activities were assessed with a dual-luciferase assay kit. Briefly, cells were washed once with 1× PBS and lysed with 100 μl of ice-cold reporter lysis buffer. Ten microlitres of cell lysate were then added to 50 μl of the luciferase substrate solution, following which 50 μl of stop and glow buffer was added for Renilla reading. Bioluminescence generated was measured using a Sirius luminometer (Berthold, Munich, Germany). The luminescence readings obtained were normalized to the protein content of the corresponding cell lysate.

### MTT (3-(4, 5-Dimethylthiazol-2-yl)-2, 5-diphenyltetrazolium bromide) Assay

Cell number after drug treatment was assessed by 3-(4,5-Dimethylthiazol-2-yl)-2,5-diphenyltetrazolium bromide MTT assay. The assay is based on the ability of mitochondrial dehydrogenase enzyme from viable cells to cleave the tetrazolium rings of the pale yellow MTT and form dark blue formazan crystals, which are largely impermeable to cell membranes, thus resulting in its accumulation within healthy cells. 5 mg/ml MTT was first dissolved in PBS and filter sterilized. Three hours before the end of drug incubation, MTT solution was added into each well at 1:10 dilution and incubated at 37 °C with 5% CO_2_ for 3 h. The medium was then removed prior to the addition of DMSO. The number of surviving cells is directly proportional to the amount of formazan present in the cell lysate, which was determined by measuring its absorbance at 550 nm using the Spectrofluoro Plus spectrophotometer (TECAN, GmbH, Grödig, Austria).

### Annexin V/PI assay

An early indicator of apoptosis is the rapid translocation and accumulation of the membrane phospholipid phosphatidylserine from the cytoplasmic interface to the extracellular surface. This loss of membrane asymmetry can be detected by utilizing the binding properties of Annexin V. To identify apoptosis, we stained cells with Annexin V antibody conjugated with FITC fluorescence dye. Briefly, 5 × 10^5^ cells were trypsinized, pelleted, and then stained with Annexin V-FITC conjugate. Cells were washed in PBS, resuspended in 100 μl of binding buffer containing FITC-conjugated anti-Annexin V antibody, and then analyzed using a flow cytometer (BD FACSCalibur, BD Biosciences, US).

### Tube formation assay

A total of 50,000 HUVECs were seeded onto Matrigel in complete medium in 24-well plates in triplicate and then incubated at 37 °C for 30 min. After the cells had attached to the Matrigel, media containing the drugs were added to each well. Cells were allowed to grow for 12 h. For fluorescence imaging of tubes, cells were incubated with 2 μg/ml of calcein for 30 min at 37 °C in the dark. Tubules were visualized by fluorescence and light microscopy at low magnification (×100), and representative images were captured at randomly selected microscope fields. The number of tubes and branches formed were counted during analysis.

### Immunoprecipitation

For detection of acetylation of PPARγ, immunoprecipitation with PPARγ was performed. Cells were seeded at 0.4 × 10^5^ million density and transfected with FLAG-tagged PPARγ before treatment with LBH589. Cells were harvested and cell pellets were lysed in immunoprecipitation (IP) lysis buffer (pH 8). Two hundred and fifty micrograms of proteins were pre-cleared with magnetic G beads (Millipore, Bedford, MA) for 1 h before rotating with 1 μg of FLAG antibody or normal rabbit IgG overnight at 4 °C. Magnetic G beads were added to the mixture the next day and rotated for 1.5 h. Magnetic-enriched IP samples were washed four times in cold wash buffer (200 mM Tris [pH 8.0], 100 mM NaCl, 0.5% NP-40, 2 mM DTT, 0.5 mM PMSF, 1 mM sodium orthovanadate, 1 μg/ml leupeptin, and 1 μg/ml aprotinin) at 10 min intervals. Samples were then boiled in 5× SDS loading buffer and subjected to SDS-PAGE. Proteins were then transferred to PVDF membranes and detected for acetyl-lysine.

### Data preprocessing of Affymetrix microarray gene expression

Microarray data of human breast cancer on Affymetrix U133A or U133Plus2 platforms were downloaded from Array Express and Gene Expression Omnibus (GEO). The panel of human breast cancer data utilized for analysis comprised 3992 tumor samples from 26 cohorts, including E-TABM-158 (*n* = 130), GSE11121 (*n* = 200), GSE12276 (*n* = 204), GSE1456 (*n* = 159), GSE1561 (*n* = 49), GSE19615 (*n* = 115), GSE20181 (*n* = 176), GSE2034 (*n* = 286), GSE21653 (*n* = 266), GSE23177 (n = 116), GSE23593 (*n* = 50), GSE23988 (*n* = 61), GSE25066 (*n* = 508), GSE26639 (*n* = 226), GSE31519 (*n* = 67), GSE3494 (*n* = 251), GSE3744 (*n* = 47), GSE4922 (*n* = 40), GSE5327 (*n* = 58), GSE5460 (*n* = 127), GSE5764 (*n* = 10), GSE6532 (*n* = 414), GSE6596 (*n* = 24), GSE7390 (*n* = 198), GSE9195 (*n* = 77), and HESS cohort (*n* = 133). Out of the 3992 tumor samples, 2667 samples were on the U133A platform whereas 1325 samples were on the U133Plus2 platform, and 974 had overall survival information, while 2333 had relapse-free survival information. Robust Multichip Average (RMA) normalization was performed on each dataset. The normalized data were combined and subsequently standardized using ComBat to remove batch effect [52–55]. Pre-processed normalized data of PPARγ gene expression and corresponding clinical data were extracted from GSE16391, GSE46222 and GSE147271 on GEO. Anova and paired *t*-tests were conducted using Graphpad Prism version 6.07.

### Identification of breast cancer subtypes

The breast cancer subtype signature was obtained from Prat et al., 201023. The breast cancer subtype of each sample was then predicted using the enrichment score of the breast cancer subtype signature computed by Single Sample Gene Set Enrichment Analysis (ssGSEA) [22]. Each sample was then assigned to be the subtype that had the maximum ssGSEA enrichment score.

### Orthotopic mouse model of the mammary fat pad

Female NCr nude mice (CrTac:NCr-Foxn1nu) aged 7 weeks were purchased from InVivos, Singapore. Mice were fed on a normal rodent diet and provided water ad libitum under humidity and the climate-controlled environment with a 12 h light-dark cycle. Mice were acclimatized to laboratory conditions for a minimum of 3 days prior to study initiation. All surgical procedures were performed under strict aseptic conditions according to the protocol approved by the SingHealth Institutional Animal Care and Use Committee (IACUC). Mice were anesthetized by exposure to 2.5% of isoflurane (IsoFlo, Abbott, Australia). A 1-cm incision was made at the abdominal mammary fat pad area. The growth of the tumor was induced by direct implantation of 3 × 106 luciferase-expressing MDA-MB-231 cells suspended in 30 µl of PBS/Matrigel (1:1) into the mammary fat pad tissues. The incision was closed with wound clips (Stoelting Co, USA). To relieve post-surgical pain and prevent dehydration following surgery, 1 ml of 2 mg/ml meloxicam (Troy Ilium, Australia), diluted in 0.9% saline (B. Braun, Germany) was administered subcutaneously at the right flank of mice after surgery. Mice were recovered on a heating pad until fully awake from anesthesia before being returned to cages.

### Pharmacokinetics study and analysis of LBH589

The pharmacokinetics study of LBH589 on six-week-old female NCr nude mice was approved by the SingHealth IACUC. Twenty-four female mice were housed in a controlled environment (21–24 °C; 54–75% relative humidity) with a 12-h light/dark cycle. The mice (*n* = 3 for each time point) were given intraperitoneal (i.p.) administration of LBH589 at a dose of 1, 2.5, and 5 mg/kg. Serial blood samples were collected before dosing and at 10 min, 30 min, 1, 2, 4, 6, and 8 h post-dose from the facial vein. After centrifuging at 14,000 rpm (4 °C) for 6 min, the serum was collected and stored at −80 °C till analysis.

LBH589 concentration was determined by a validated LC-MS/MS assay using vorinostat-d5 as the internal standard. The LC-MS/MS system consisted of an Agilent 1100 binary pump connected to an API 4000 triple-quadrupole mass spectrometer. Chromatographic separations were achieved on an Agilent ZORBAX Eclipse XDB C8 column (2.1 mm × 50 mm, 5 µm). Mobile phase solvent A was 0.1% formic acid and solvent B was acetonitrile with a gradient elution mode. The run time was 5 min at a constant flow rate of 0.45 ml/min. LC-MS/MS was carried out under positive electrospray ionization (ESI) and multiple reaction monitoring (MRM) mode. Mass transitions of LBH589 and vorinostat-d5 were 350 > 158 and 270 > 237, respectively. SCIEX Analyst software (version 1.4.2) was used for data acquisition and analysis. Good linearity was achieved for LBH589 in the range of 5–1000 ng/mL with *R*^2^ (coefficient of determination) >0.998. Pharmacokinetic calculations were performed using WinNonLin version 5.3.

### Bioluminescent Imaging

Tumor growth was screened by imaging from the ventral view using the Xenogen IVIS system (Caliper Life Sciences, CA, USA). D-luciferin (Gold Bio, USA) at 150 mg/kg in phosphate-buffered saline was injected i.p. into the mice 10 min before imaging. Mice were anesthetized with 2.5% isoflurane. After 10 min, they were placed on the warmed stage inside the light-tight camera box with continuous exposure to 1% isoflurane. Imaging time was 1 min/mouse. Bioluminescent signals around the tumor sites were identified and quantified as total photon counts using Living Image 4.2 software (Xenogen). When the bioluminescent signal reached 107, mice were randomly distributed into different experimental groups.

### Toxicity study

A total of 39 mice were randomly assigned to 5 treatment groups with at least 7 animals in each group. Mice were treated with vehicle (10% DMSO), 2.5 mg/kg or 7.5 mg/kg of LBH589, 10 mg/kg, or 20 mg/kg of Rosiglitazone via i.p. injection five days per week. The animals were evaluated for changes in clinical signs (stooling, activity level, and body weight). Bodyweight was measured prior to dosing and mice were monitored regularly for any signs of distress after drug administration. All mice were humanely euthanized after 24 days.

### Experimental study

Mice injected with MDA-MB-231 cells expressing luciferase were randomly assigned to receive vehicle (10% DMSO), 2.5 mg/kg of LBH589, 10 mg/kg of Rosiglitazone, or a combination of LBH589 and Rosiglitazone via i.p. injection five days per week. Each treatment group consisted of at least 7 mice. The bodyweight of mice was measured prior to drug administration. Tumor growth was monitored twice a week using the Xenogen IVIS system throughout the entire 28 days of treatment. At the end of the experiment, primary mammary tumors were first imaged in vivo, and tumors were excised from the mice. Half of the tumor from each mouse was subsequently fixed in formaldehyde buffer solution (4% w/w formaldehyde with 1.16% w/v phosphate buffer) (Integrated Contract Manufacturing, Singapore) at room temperature for 24 h and replaced with phosphate-buffered saline until further processing. The other half of the tumor was immediately snapped-frozen in liquid nitrogen and stored at −80 °C until further analysis.

### Histopathological analysis and clinical correlations of PPARγ and HDAC1/2 expression in human invasive ductal carcinoma specimens

Tissue microarray (TMA) slides of invasive ductal carcinoma (IDC) cases were obtained from the Department of Pathology, Singapore General Hospital. The TMA samples consisted of 390 IDC patients and 60 normal breast tissues. Clinicopathological characteristics were retrieved for statistical analyses, including patient’s age, race, tumor size, histological grade of the tumor, lymphovascular invasion, axillary lymph nodes status, tumor type, estrogen receptor status, progesterone receptor status, and HER2 receptor status. Ethics approval for the study was obtained from the Institutional Review Board, Singapore General Hospital.

Anti-HDAC1 (Proteintech), HDAC2 (Proteintech), and PPARγ (Proteintech) antibodies were used for the immunohistochemical staining of the TMA sections as described previously58–60. Briefly, the TMA sections were de-paraffinized in clearance and rehydrated through a graded series of ethanol. Endogenous peroxidase activity was quenched with 3% hydrogen peroxidase for 30 min and antigen retrieval was performed through boiling in 10 mM citrate buffer (pH 6.0) for 20 min in a microwave oven. Next, blocking with goat serum was carried out for 1 h prior to overnight incubation at 4 °C with the HDAC1 antibody (1:100 dilution), HDAC2 antibody (1:50 dilution), or PPARγ (1:30 dilution). A secondary antibody (DAKO Envision Kit) was then added and incubated for 1 h at room temperature. The sections were visualized using diaminobenzidine as the substrate and counterstained using Shandon’s haematoxylin.

The intensity of the nuclear staining (HDAC1 and HDAC2) or both nuclear and cytoplasmic staining (PPARγ) in the ductal tissues was graded as absent (0), weak (1+), moderate (2+), or strong (3+) by two independent blinded observers. The percentage of cells positively stained was recorded as the total percentage score (TPS). The statistical analysis was performed using the PASW Statistics 18 software (SPSS). Correlations between the expression level of HDAC1, HDAC2, and PPARγ with the clinicopathological parameters were determined using the Fisher’s exact and Kendall-tau tests. A *p*-value below 0.05 was considered statistically significant.

### Chick chorioallantoic membrane (CAM) and tumor cell inoculation

Fertilized chicken eggs (Bovans Goldline Brown) were purchased from Chew’s Agriculture Pte Ltd and placed horizontally in a 37.5 °C incubator with 70% humidity on Embryonic Day 0. On Embryonic Day 3, a sharp weighted tool was used to poke a hole at the apex of the eggshell, and 3 mL of albumin was removed using a 5 ml syringe and 18G needle in order to drop the chick chorioallantoic membrane (CAM). The sharp-weighted tool was then used to poke a hole in the middle of the egg before using curved scissors to cut a 1cm2 hole. The eggs were screened, and dead embryos were removed. The hole was then sealed with the 1624W Tegaderm semi-permeable membrane and placed back into the incubator.

On Embryonic Day 7, MDA-MB-231 cells were prepared for inoculation onto the CAM. DMEM was aspirated, cells were washed with PBS before the addition of 1 ml of 2.5 g/l Trypsin/EDTA solution onto the culture dish. The cells were then removed into a 15 ml Falcon tube and centrifuged for 3 min at 1200 rpm, before the supernatant was removed. Matrigel was added to the cell pellet on ice to resuspend the pellet, before the addition of 2 × 106 cells in a 50 µl of Matrigel-cell mixture onto CAM. The hole was then re-sealed with the Tegaderm semi-permeable membrane.

### Ultrasound imaging and drug treatment

On Embryonic Day 10, the Tegaderm membrane was removed and Aquasonic gel was added onto the cling wrap which was carefully placed over the CAM. Using Visualsonics Vevo 2100 Imaging system, a 550D transducer connected to a 3D acquisition motor was used to obtain ultrasound images of the tumors formed on the CAM. Parallel 2D sections obtained were further reconstructed to form 3D images of the tumors. Tumor volumes and percentage vasculature were calculated by the Vevo Lab 1.7.0 programme.

Twenty microliters of single or combination treatments of LBH589 and rosiglitazone were added onto a 3.5 mm diameter autoclaved filter paper placed next to the formed tumor on the CAM. The drug was topped up every 24 h to prevent drying up of the filter paper. A final ultrasound scan was performed after 48 h addition of the drugs. Percentage changes were calculated based on before and after drug treatment.

### Statistical analysis

Statistical analysis was performed using paired Student’s *t*-test, Mann-Whitney test, and Spearman correlation test as appropriate in Matlab®, and two-sided nominal *p*-values of less than 0.05 were considered significant. One-way ANOVA with Tukey Post-hoc analysis was used for multiple group statistical comparisons. Statistical significance evaluation for bioinformatics data is as described in the preceding paragraphs.

## Supplementary information

Supplementary Figure Legends

Supplementary Table 1

Figure S1

Figure S2

Figure S3

Figure S4

Figure S5

Figure S6

Figure S7

Figure S8

## Data Availability

Microarray data of human breast cancer on Affymetrix U133A or U133Plus2 platforms were downloaded from Array Express and Gene Expression Omnibus (GEO). Pre-processed normalized data of PPARγ gene expression and corresponding clinical data were extracted from GSE16391, GSE46222, and GSE147271 on GEO. Source data supporting the findings of this study are available from the corresponding author on request.
