# Supplementary material for: Epigenetic derepression converts PPARγ into a druggable target in triple-negative and endocrine-resistant breast cancers
Source: Cell Death Discov. 2021 Sep 27;7:265. doi: 10.1038/s41420-021-00635-5 (PMC8476547; doi:10.1038/s41420-021-00635-5)
Supplement: Supplementary file 1 — Supplementary Figure Legends [file 41420_2021_635_MOESM1_ESM.docx]

**SUPPLEMENTARY Figure Legends**

**Supplementary Figure S1**

(A) Kaplan Meier plots for 15 breast carcinoma gene expression datasets with available data on *PPARG* expression and overall survival. PPARG expression were dichotomized by study-specific medians. Median values of ln (PPARG expression+1), hazard ratios (95% CI), and log-rank P values are given within each panel. The PubMed ID (PMID) corresponding to the studies associated with each gene expression dataset are shown above the Kaplan Meier plots.

**Supplementary Figure S2**

(A) Random-effects and inverse variance-weighted meta-analysis using hazard ratio estimates from Supplementary Figure S1. The pooled hazard ratio for overall survival is 0.84 (95% CI: 0.71-0.99; P=0.036) in favor of high *PPARG* expression, based on 2,151 breast cancer patients across 15 published datasets.

**Supplementary Figure S3**

(A) Left: HDAC1 immunostaining in breast tissues showing nuclear localization. A higher percentage of cells stained with HDAC1 was observed in the IDC case of tumor size >20mm compared to the IDC case having tumor size ≤20mm. Right: Statistics of HDAC1 expression between IDC tissues of tumor sizes ≤20mm and >20 mm.

(B) Left: HDAC2 immunostaining in breast tissues showing nuclear localization. A higher percentage of cells stained with HDAC2 was observed in the IDC case of tumor size> 20 mm compared to the IDC case having tumor size ≤ 20 mm. Right: Statistics of HDAC2 expression between IDC tissues of tumor sizes ≤20mm and >20 mm.

**Supplementary Figure S4**

1. Western blot analysis of MDA-MB-231 cells treated with 0, 1, 3, 5, 10, and 20µM SAHA for 24h.
2. Western blot analysis of MDA-MB-231 cells treated with 0, 5, 10, 20, and 40µM Droxinostat for 24h.

**Supplementary Figure S5**

1. The figure depicts the impact of two drugs Actos (PPARγ ligand) and Panobinostat (LBH589) individually and in combination on the tumor phenotypes: Tumor Volume, Proliferation, Viability, and Angiogenesis.
2. Predictive in silico Tumor Cell platform generated results: Reduction in biomarkers-CCND1, ERK, STAT3, AKT, BCL2, VEGFA, and survivin with Panobinostat and Actos tested individually and in combination.
3. T47D cells were subjected to 50nM LBH589, 20µM ciglitazone, 60µM rosiglitazone, or a combination of LBH589 and ciglitazone/ rosiglitazone for 24h. RT-PCR analysis of relative *Cyclin D1* mRNA levels normalized to 18S mRNA levels.
4. RT-PCR analysis of relative *BIRC* mRNA levels normalized to 18S mRNA levels.
5. RT-PCR analysis of relative *MnSOD* mRNA levels normalized to 18S mRNA levels.
6. RT-PCT analysis of relative *PTEN* mRNA levels normalized to 18S mRNA levels. Values represent mean ± SD, n=3, *P<0.05 vs. control, **P<0.005 vs. control
7. The number of tubes was counted from the tube formation assay of HUVECs treated with 50nM LBH589, 20µM ciglitazone, 60µM rosiglitazone, or a combination of LBH589 and ciglitazone/ rosiglitazone for 12h. Values represent mean ± SD, n=3, *P<0.05 vs. control.
8. The number of branching points was counted from the tube formation assay of HUVECs treated with 50nM LBH589, 20µM ciglitazone, 60µM rosiglitazone, or a combination of LBH589 and ciglitazone/ rosiglitazone for 12h. Values represent mean ± SD, n=3, *P<0.05 vs. control.
9. The number of tubes was counted from the tube formation assay of HUVECs treated with a combination of LBH589 and ciglitazone/rosiglitazone for 12h +/- 4h pre-incubation of 10µM GW9662. Values represent mean ± SD, n=3, *P<0.05 vs. control.
10. The number of branching points was counted from the tube formation assay of HUVECs treated with a combination of LBH589 and ciglitazone/rosiglitazone for 12h +/- 4h pre-incubation of 10µM GW9662. Values represent mean ± SD, n=3, *P<0.05 vs. control.

**Supplementary Figure S6**

1. PPARγ activity of MCF-7 and MDA-MB-231 cells treated with 50nM LBH589, 20µM ciglitazone, or a combination of both for 16h +/- 4h pre-incubation of 10µM GW9662. Values represent mean ± SD, n=3, *P<0.05 vs. control.
2. Annexin V/PI staining assay of MCF-7 and MDA-MB-231 cells treated with 50nM LBH589, 20µM ciglitazone, or a combination of both for 24h. Values represent mean ± SD, n=3, *P<0.05 vs. control.
3. PPARγ activity of MCF-7 and MDA-MB-231 cells transfected with control shRNA and PPARγ shRNA and treated with 50nM LBH589, 20µM ciglitazone, or a combination of both for 16h. Values represent mean ± SD, n=3, *P<0.05 vs. control.
4. Cell viability assay of MCF-7 and MDA-MB-231 cells treated with 50nM LBH589, 20µM ciglitazone, or a combination of both for 24h +/- 4h pre-incubation of 10µM GW9662. Values represent mean ± SD, n=3, *P<0.05 vs. control.
5. Annexin V/PI staining assay of MDA-MB-231 cells treated with a combination of LBH589 + ciglitazone or LBH589 + rosiglitazone for 24h +/- 4h pre-incubation of 10µM GW9662.
6. Annexin V/PI staining assay of MCF-7 cells treated with a combination of LBH589 + ciglitazone or LBH589 + rosiglitazone for 24h +/- 4h pre-incubation of 10µM GW9662.
7. Cell viability assay of MCF-7 and MDA-MB-231 cells transfected with control shRNA and PPARγ shRNA and treated with 50nM LBH589, 20µM ciglitazone, or a combination of both for 16h. Values represent mean ± SD, n=3, *P<0.05 vs. control.
8. Annexin V/PI staining assay of MDA-MB-231 cells transfected with pcmx or pcmx-DN PPARγ and treated with a combination of LBH589 + ciglitazone or LBH589 + rosiglitazone for 24h.
9. Annexin V/PI staining assay of MCF-7 cells transfected with pcmx or pcmx-DN PPARγ and treated with a combination of LBH589 + ciglitazone or LBH589 + rosiglitazone for 24h.

**Supplementary Figure S7**

1. PPARγ activity of MDA-MB-231 cells treated with 3µM SAHA, 20µM ciglitazone, 60µM rosiglitazone, or a combination of SAHA + ciglitazone / rosiglitazone for 16h. Values represent mean ± SD, n=3, *P<0.05 vs. control.
2. PPARγ activity of MDA-MB-231 cells treated with 20µM Droxinostat, 20µM ciglitazone, 60µM rosiglitazone, or a combination of DROX + ciglitazone / rosiglitazone for 16h. Values represent mean ± SD, n=3, *P<0.05 vs. control.
3. Cell viability assay of MDA-MB-231 cells treated with 3µM SAHA, 20µM ciglitazone, 60µM rosiglitazone, or a combination of SAHA + ciglitazone / rosiglitazone for 24h. Values represent mean ± SD, n=3, *P<0.05 vs. control.
4. Cell viability assay of MDA-MB-231 cells treated with 20µM Droxinostat, 20µM ciglitazone, 60µM rosiglitazone, or a combination of DROX + ciglitazone / rosiglitazone for 24h. Values represent mean ± SD, n=3, *P<0.05 vs. control.
5. PPARγ activity of T47D A18 and T47D A18 4OHT cells treated with 50nM LBH589, 20µM ciglitazone, or a combination of both for 24h. Values represent mean ± SD, n=3, *P<0.05 vs. control.
6. PPARγ activity of WS8 and ICI-R cells treated with 50nM LBH589, 20µM ciglitazone, or a combination of both for 24h. Values represent mean ± SD, n=3, *P<0.05 vs. control.
7. Cell viability assay of T47D A18 and T47D A18 4OHT cells treated with 50nM LBH589, 20µM ciglitazone, or a combination of both for 24h. Values represent mean ± SD, n=3, *P<0.05 vs. control.
8. Cell viability assay of WS8 and ICI-R cells treated with 50nM LBH589, 20µM ciglitazone, or a combination of both for 24h. Values represent mean ± SD, n=3, *P<0.05 vs. control.

**Supplementary Figure S8**

(A) Western blot analysis of MCF-10A and MCF-12A treated with 0, 50, and 100nM LBH589 for 24h.

(B) PPARγ activity of MCF-10A and MCF-12A cells treated with 0, 50, and 100nM LBH589 for 24h. Values represent mean ± SD, n=3.

(C) Cell viability assay of MCF-10A and MCF-12A cells treated with 50nM LBH589, 20µM ciglitazone, or a combination of both for 24h. Values represent mean ± SD, n=3.

(D) Cell viability assay of MCF-10A and MCF-12A cells treated with 50nM LBH589, 60µM rosiglitazone, or a combination of both for 24h. Values represent mean ± SD, n=3.

(E) Light microscopy images of MCF-10A treated with 50nM LBH589, 20µM ciglitazone, 60µM rosiglitazone, or a combination of LBH589 and ciglitazone/ rosiglitazone for 24h (x20 magnification).

(F) Light microscopy images of MCF-12A treated with 50nM LBH589, 20µM ciglitazone, 60µM rosiglitazone, or a combination of LBH589 and ciglitazone/ rosiglitazone for 24h (x20 magnification).
