# Supplementary material for: Epigenetic derepression converts PPARγ into a druggable target in triple-negative and endocrine-resistant breast cancers
Source: Cell Death Discov. 2021 Sep 27;7:265. doi: 10.1038/s41420-021-00635-5 (PMC8476547; doi:10.1038/s41420-021-00635-5)
Supplement: Supplementary file 2 — Supplementary Table 1 [file 41420_2021_635_MOESM2_ESM.docx]

**SUPPLEMENTARY TABLES**

Table S1. Combination indexes (CI) of LBH589 and PPARγ ligands in breast cancer cell lines.

a: Computed by CompuSyn software. CI < 1.0 indicates synergism.

| Cell line | Drug combination | Ratio of LBH589 to PPARγ ligand | % kill | CI at % kill^a^ |
| --- | --- | --- | --- | --- |
| MCF7 | LBH589 + ROSIG | 1:2000 | 60.0 | 0.82913 |
| MDA-MB-231 |  |  | 70.0 | 0.61927 |
| T47D A18 |  |  | 60.0 | 0.82913 |
| T47D A18-4OHT |  |  | 55.0 | 0.95031 |
| WS8 |  |  | 55.0 | 0.95031 |
| MCF7-ICIR |  |  | 60.0 | 0.82913 |
| MCF7 | LBH589 + CIG | 1:667 | 50.0 | 0.74652 |
| MDA-MB-231 |  |  | 60.0 | 0.50696 |
| T47D A18 |  |  | 45.0 | 0.90507 |
| T47D A18-4OHT |  |  | 45.0 | 0.90507 |
| WS8 |  |  | 55.0 | 0.61618 |
| MCF7-ICIR |  |  | 50.0 | 0.74652 |
