# Supplementary figures and images for: Epigenetic derepression converts PPARγ into a druggable target in triple-negative and endocrine-resistant breast cancers
Source: Cell Death Discov. 2021 Sep 27;7:265. doi: 10.1038/s41420-021-00635-5 (PMC8476547; doi:10.1038/s41420-021-00635-5)

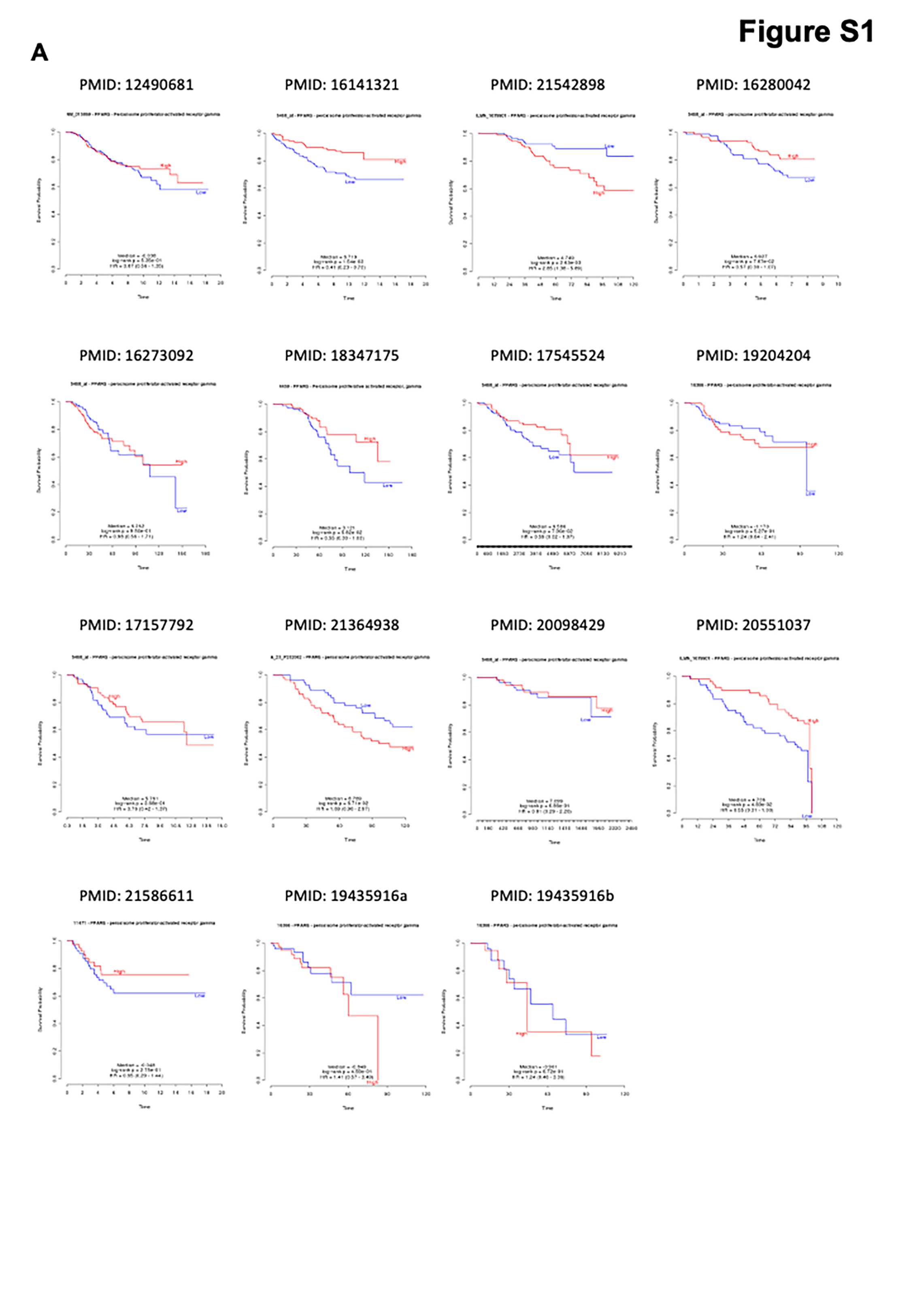

Supplement: Supplementary file 3 — Figure S1 [file 41420_2021_635_MOESM3_ESM.tif]

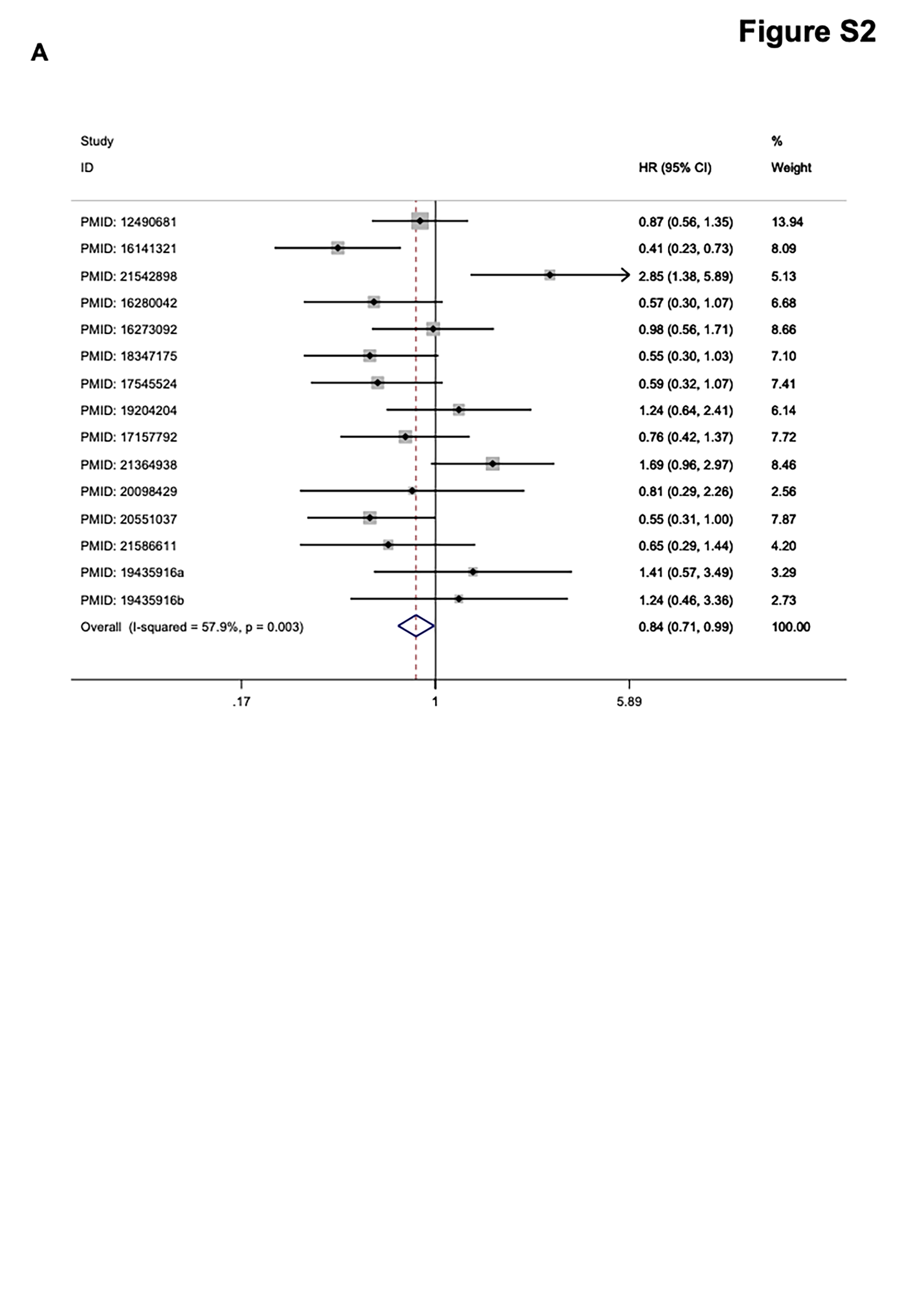

Supplement: Supplementary file 4 — Figure S2 [file 41420_2021_635_MOESM4_ESM.tif]

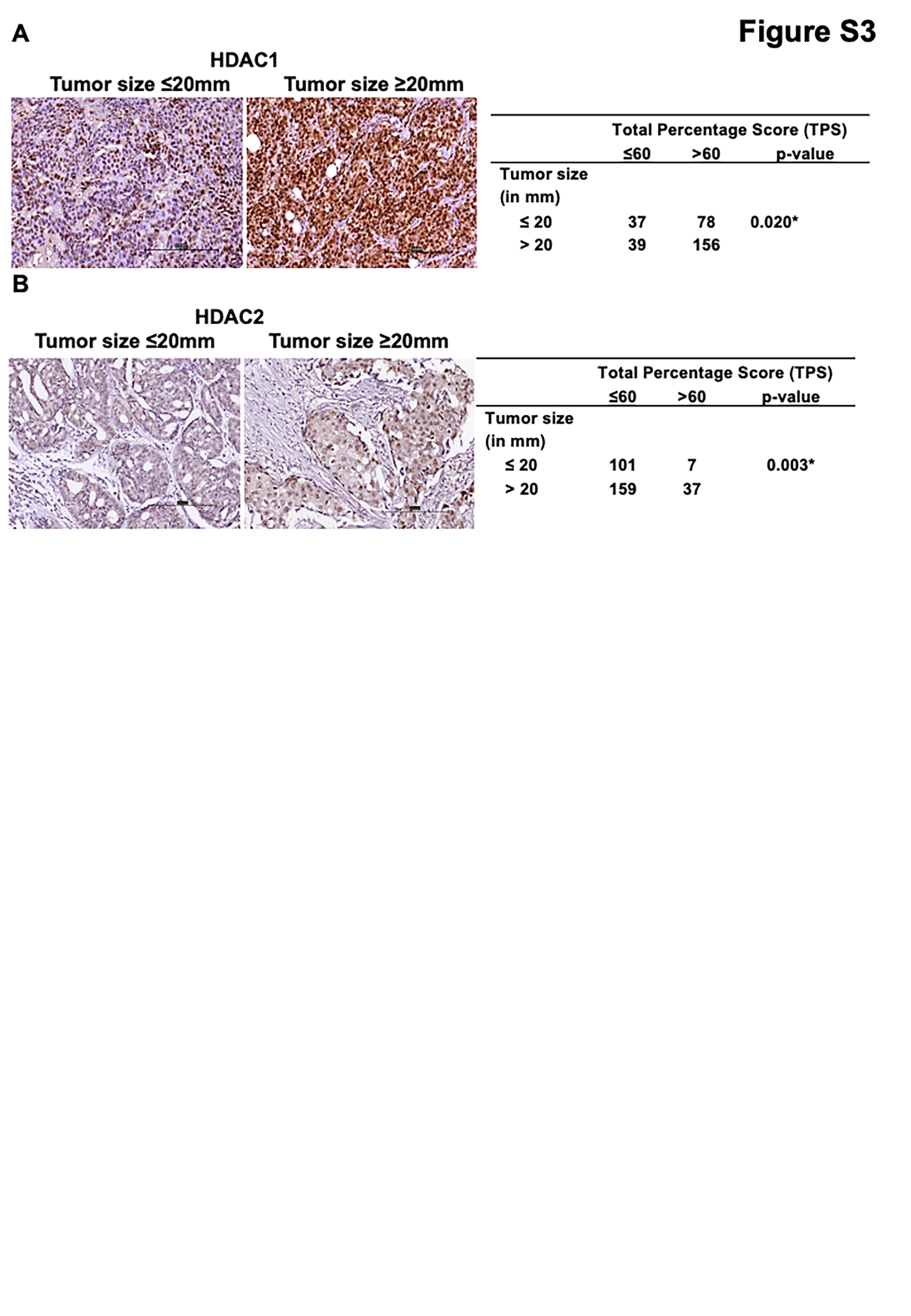

Supplement: Supplementary file 5 — Figure S3 [file 41420_2021_635_MOESM5_ESM.tif]

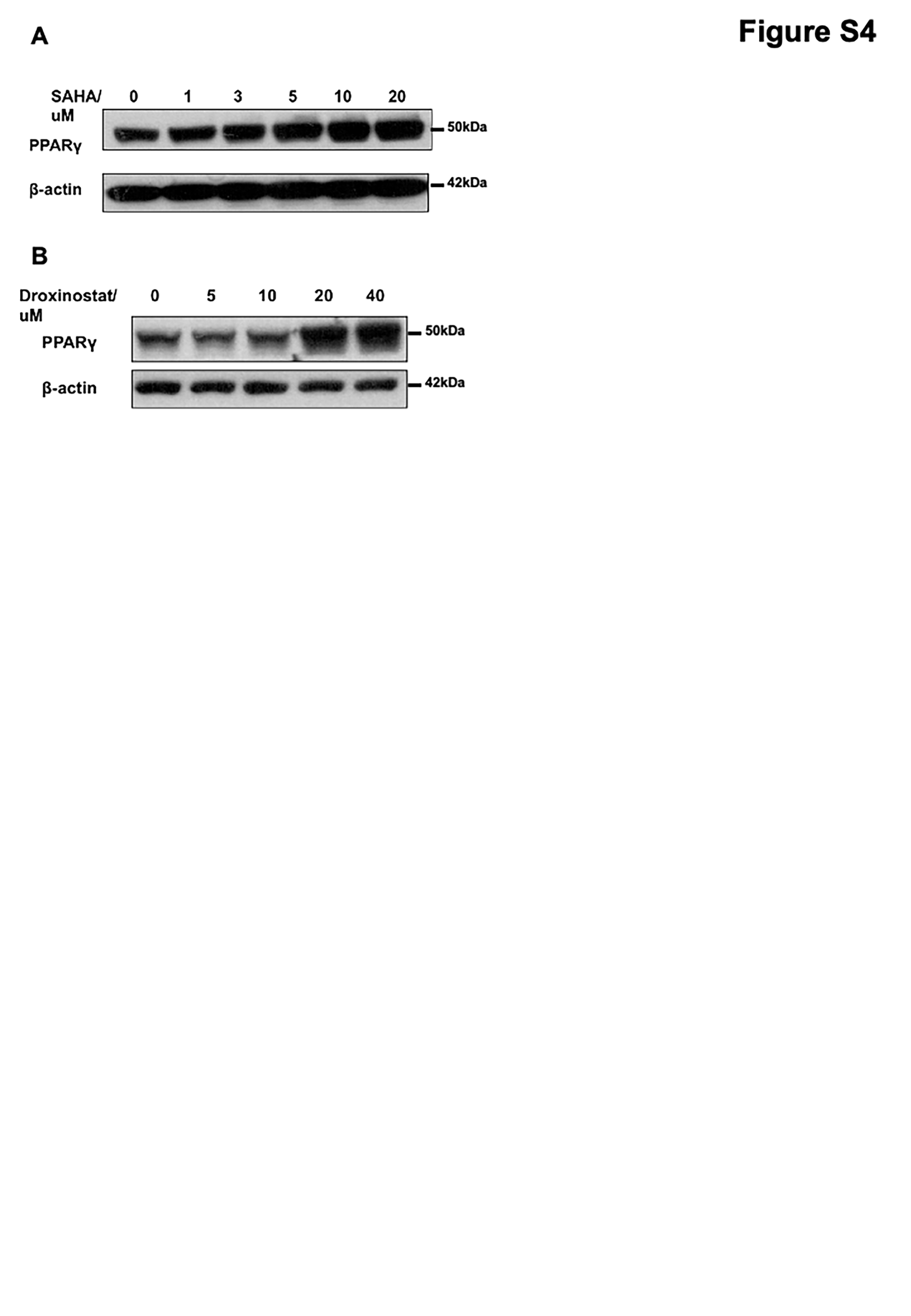

Supplement: Supplementary file 6 — Figure S4 [file 41420_2021_635_MOESM6_ESM.tif]

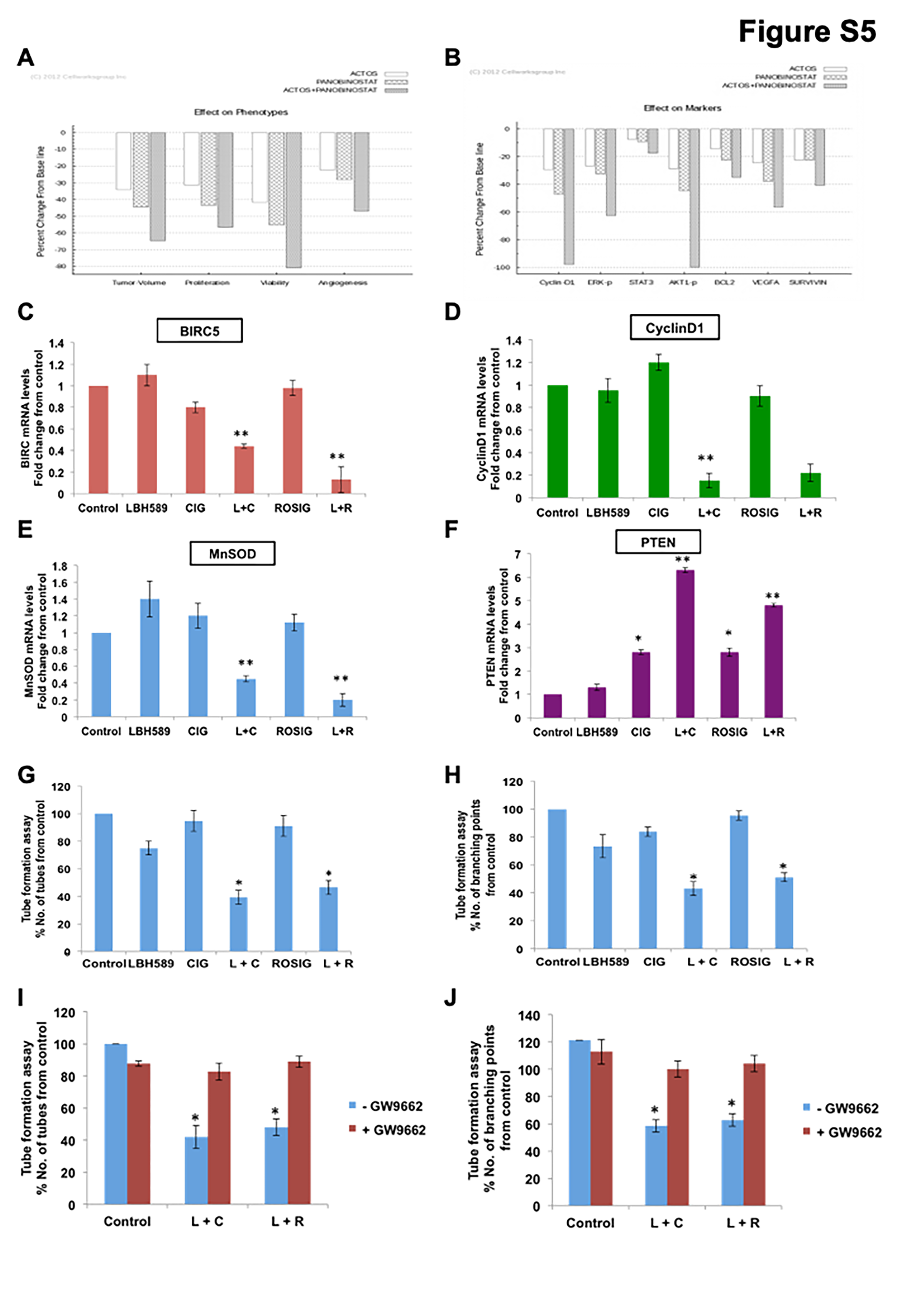

Supplement: Supplementary file 7 — Figure S5 [file 41420_2021_635_MOESM7_ESM.tif]

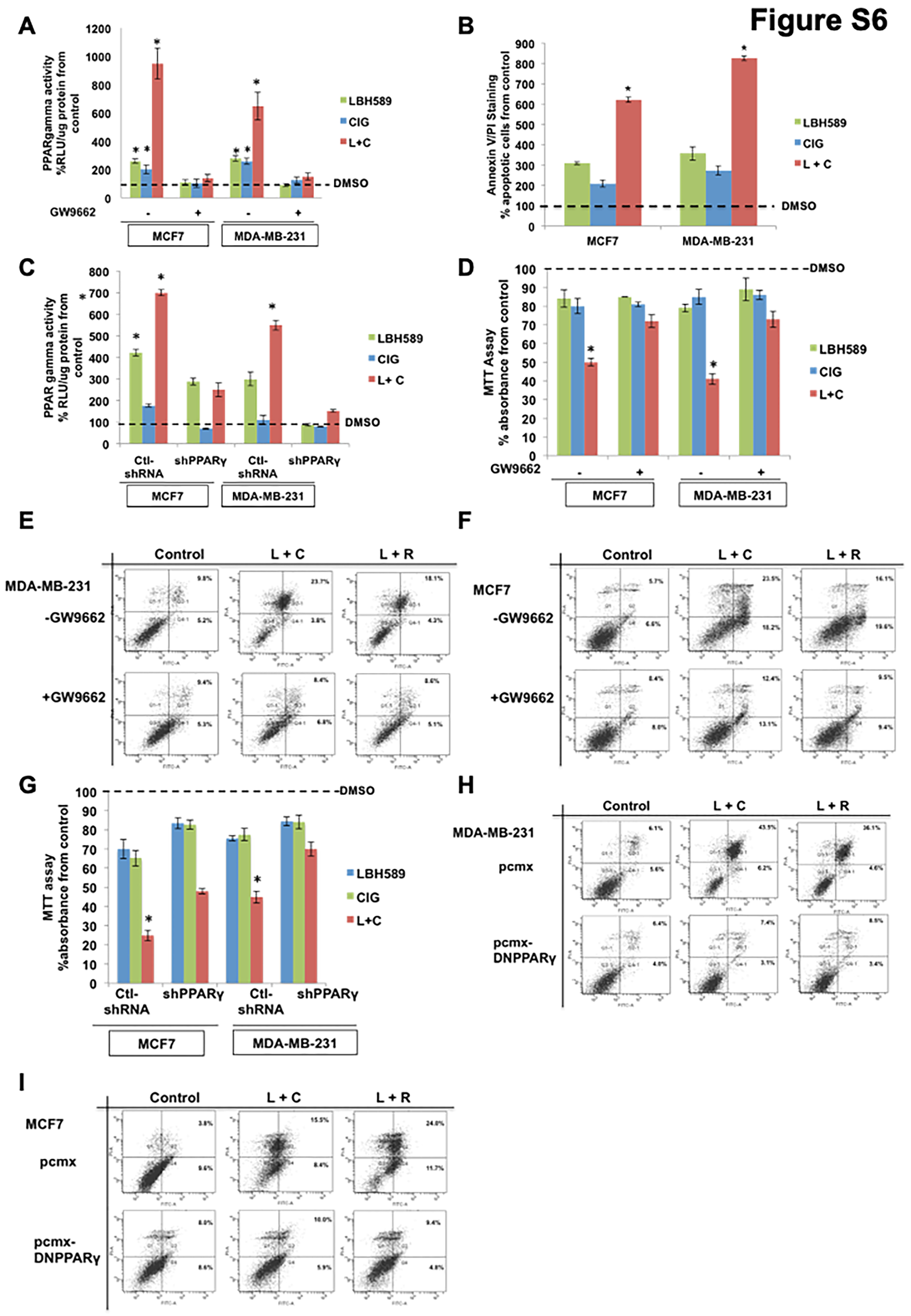

Supplement: Supplementary file 8 — Figure S6 [file 41420_2021_635_MOESM8_ESM.tif]

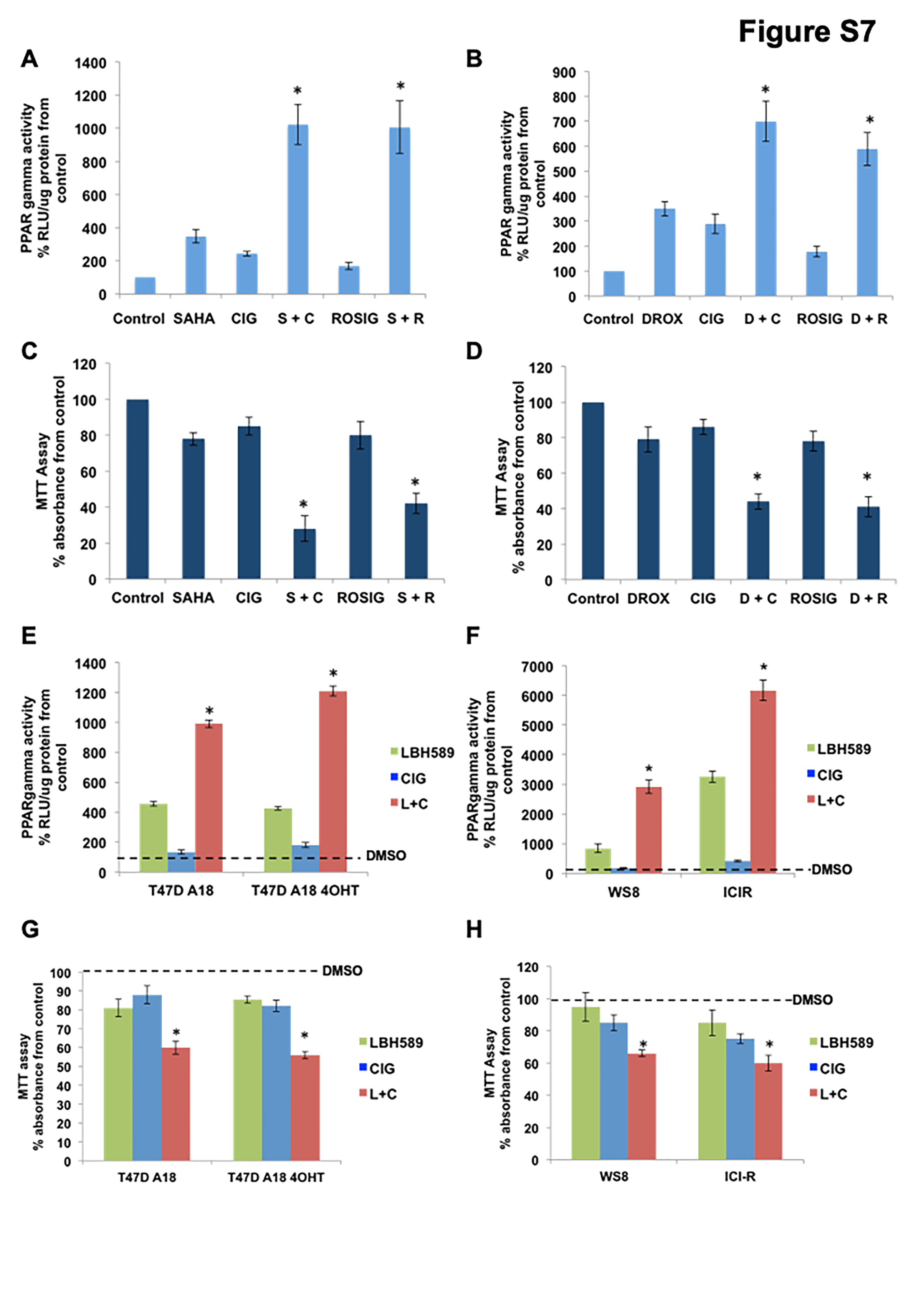

Supplement: Supplementary file 9 — Figure S7 [file 41420_2021_635_MOESM9_ESM.tif]

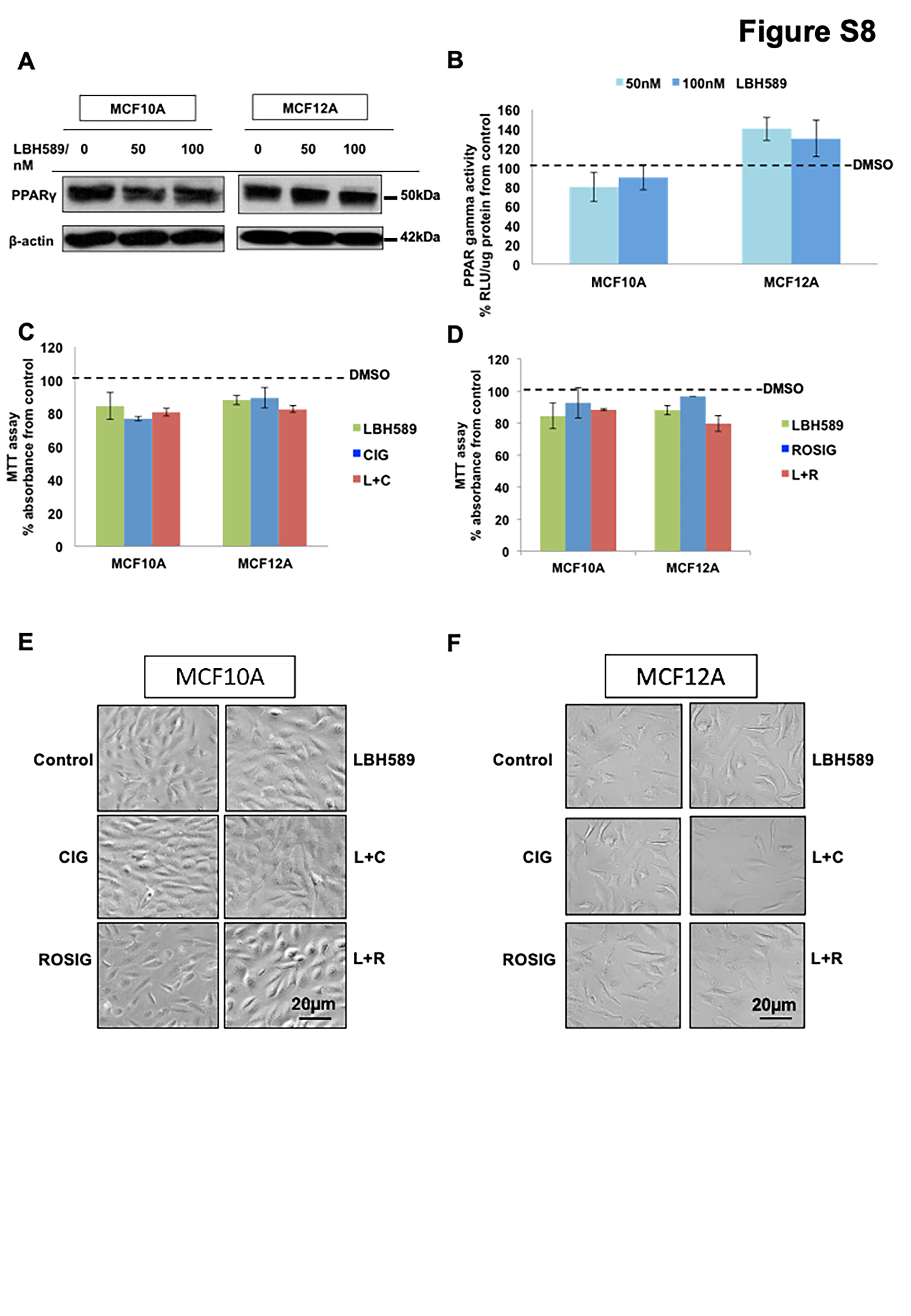

Supplement: Supplementary file 10 — Figure S8 [file 41420_2021_635_MOESM10_ESM.tif]
